# Five New Limonoids from Peels of Satsuma Orange (*Citrus reticulata*)

**DOI:** 10.3390/molecules22060907

**Published:** 2017-05-31

**Authors:** Takashi Kikuchi, Yasuaki Ueno, Yoshino Hamada, Chika Furukawa, Takako Fujimoto, Takeshi Yamada, Reiko Tanaka

**Affiliations:** Faculty of Pharmaceutical Sciences, Osaka University of Pharmaceutical Sciences, 4-20-1 Nasahara, Takatsuki, Osaka 569-1142, Japan; t.kikuchi@gly.oups.ac.jp (T.K.); e10331@gap.oups.ac.jp (Y.U.); yoshinotama@yahoo.co.jp (Y.H.); chika1026@pearl.ocn.ne.jp (C.F.); e11537@gap.oups.ac.jp (T.F.); yamada@gly.oups.ac.jp (T.Y.)

**Keywords:** limonoid, Rutaceae, satsuma orange, *Citrus reticulata*, inhibitory activity on nitric oxide production

## Abstract

Five new: 21,23-dihydro-21-hydroxy-23-oxonomilin (**1**), 21,23-dihydro-23-methoxy-21-oxonomilin (**2**), 21,23-dihydro-21-hydroxy-23-oxonomilinic acid methyl ester (**3**), 21,23-dihydro-23-methoxy-21-oxolimonin (**4**), and 21,23-dihydro-21-oxolimonin (**5**), and seven known limonoids were isolated from peels of satsuma orange (*Citrus reticulata*). The isolated compounds were evaluated for their inhibitory effects on macrophage activation by an inhibitory assay of nitric oxide (NO) production. Among them, compound (**2**) exhibited NO inhibitory activity without cytotoxicity.

## 1. Introduction

Satsuma orange (Scientific name: *Citrus reticulata* Blanco*;* Synonym: *Citrus unshiu* Marcov.; Japanese name: unshu mikan), belongs to Rutaceae, and is cultivated in Japan. The peels of its fruits, called chinpi, have been used as an aromatic stomachic, cold medicine, expectorant, and antitussive [1]. Limonoids [2,3], flavonoids [4,5,6,7], tocopherol analogues [8,9] have been isolated from peels of *C. reticulata*, phenyl glycosides from flower buds [10], and a limonoid [11], flavonoids [11], and a cyclic peptide from its fruits [12]. Moreover, some bioactivities of constituents from *C. reticulata* have been reported, such as the suppression of adipogenesis by a limonoid [3], the inhibitory activity on histamine release from rat peritoneal mast cells [4], hypotensive activity [5] and antioxidant activity [6] of flavonoids, the radical-scavenging activity [8] and hepatoprotective and neuroprotective activities [9] of tocopherol analogues, as well as antimicrobial activities of essential oil [13]. In the present study, we isolated five new limonoids **1**–**5**; 21,23-dihydro-21-hydroxy-23-oxonomilin (**1**), 21,23-dihydro-23-methoxy-21-oxonomilin (**2**), 21,23-dihydro-21-hydroxy-23-oxonomilinic acid methyl ester (**3**), 21,23-dihydro-23-methoxy-21-oxolimonin (**4**), and 21,23-dihydro-21-oxolimonin (**5**), along with known compounds (**6**–**12**). Compounds **1**–**12** were evaluated for inhibitory effects of limonoids on nitric oxide (NO) production by macrophages.

## 2. Results and Discussion

**Figure 1:**
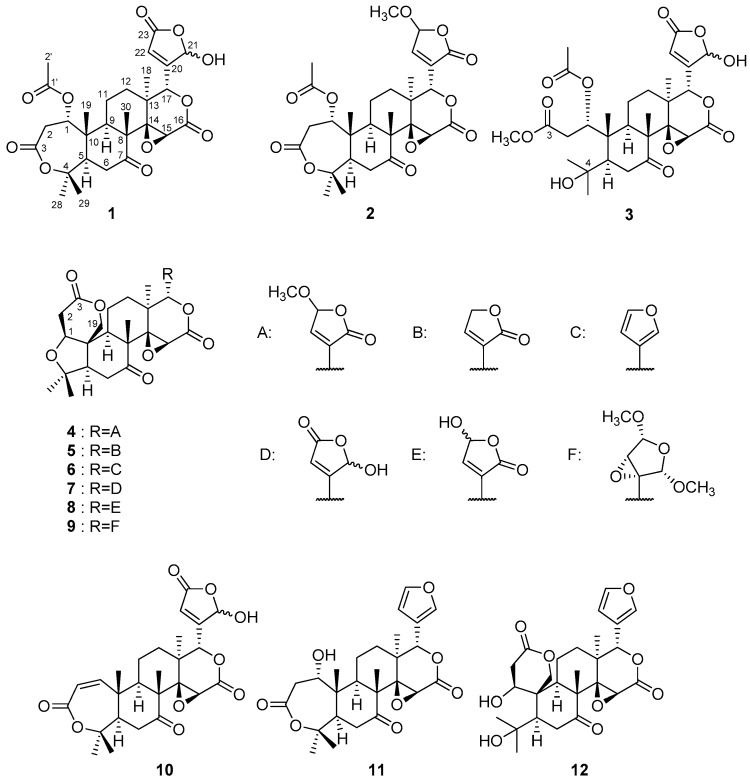
Structures of limonoids isolated from peels of *C. reticulata.*

Five new (**1**–**5**), and seven known compounds (**6**–**12**) were isolated from the *C. reticulata* peels (Figure 1). The known compounds were identified as limonin (**6**) [14,15], shihulimonin A (**7**) [16], limonexic acid (**8**) [17], evolimorutanin (**9**) [18], kihadanin A (**10**) [19], deacetylnomilin (**11**) [20], and ichangin (**12**) [21] by comparison of their spectroscopic data with those previously reported.

**Figure 2:**
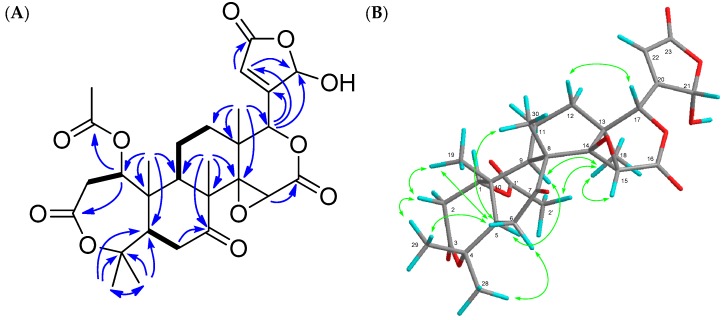
Key HMBC (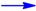) and ^1^H-^1^H COSY (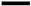) (**A**) and NOE (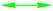) (**B**) correlations of compound **1**.

**Figure 3:**
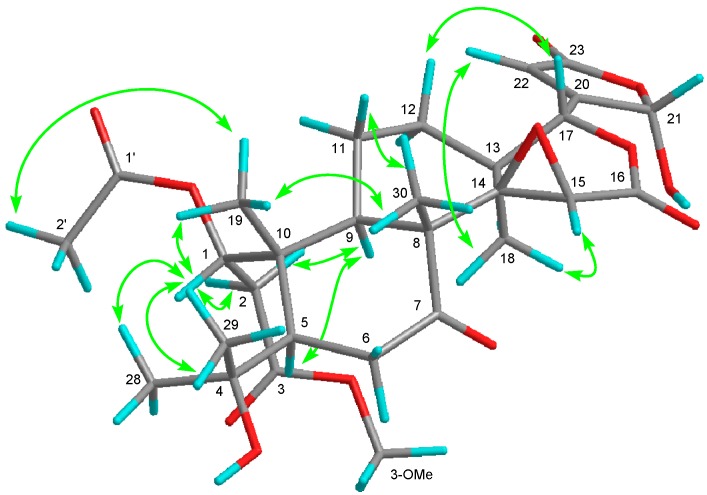
Key NOE correlations (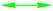) of compound **3**.

21,23-Dihydro-21-hydroxy-23-oxonomilin (**1**) was obtained as colorless crystals. Its molecular formula was established as C_28_H_34_O_11_ (*m*/*z* 547.2178 [M + H]^+^, calcd.: 547.2179) by the HRFABMS. The IR spectrum showed the presence of hydroxy (3466 cm^−1^) and carbonyl (1733 cm^−1^) groups. The ^1^H- and ^13^C-NMR spectra indicated the presence of six methyl groups observed as singlets including acetyl methyls (δ_H_ 1.11 (s), 1.16 (s), 1.34 (s), 1.48 (s), 1.56 (s), 2.10 (s)), three oxymethines (δ_H_ 3.70 (s), 5.05 (brd), 5.34 (brd); δ_C_ 52.6 (d), 70.7 (d), 78.1 (d)), two unprotonated oxycarbons (δ_C_ 65.0 (s), 84.4 (s)), a hemiacetal (δ_H_ 5.99 (brs); δ_C_ 97.4 (d)), a trisubstituted olefin (δ_H_ 6.30 (dd); δ_C_ 123.2 (d), 162.5 (s)), a ketone (δ_C_ 206.5 (s)), four ester carbonyls (δ_C_ 165.5 (s), 168.9 (s), 169.1 (s), 169.6 (s)) (Table 1). In the HMBC experiment, the following correlations were observed: Me-18 (δ_H_ 1.11 (s))/C-12, C-13, C-14 (δ_C_ 65.0 (s)), and C-17 (δ_C_ 78.1 (d)); Me-19 (δ_H_ 1.34 (s))/C-1 (δ_C_ 70.7 (d)), C-5, C-9, and C-10; Me-28 (δ_H_ 1.48 (s)) and Me-29 (δ_H_ 1.56 (s))/C-4 (δ_C_ 84.4 (s)) and C-5; Me-30 (δ_H_ 1.16 (s))/C-7 (δ_C_ 206.5 (s)), C-8, C-9, and C-14; H-1 (δ_H_ 5.05 (brd))/C-3 (δ_C_ 169.1 (s)) and 1-OCOCH_3_ (δ_C_ 169.6 (s)); H-15 (δ_H_ 3.70 (s))/C-16 (δ_C_ 165.5 (s)); H-22 (δ_H_ 6.30 (dd))/C-21 (δ_C_ 97.4 (d)) (Figure 2). In addition, a trisubstituted olefin existed at C-20 and C-22, and a hemiactal carbon existed at C-21 since HMBC correlations were observed from H-17 (δ_H_ 5.34 (brd)) to the acetal carbon (δ_C_ 97.4 (d)) and olefin carbons (δ_C_ 123.2 (d), 162.5 (s)). The above data suggested that compound **1** had a similar structure to nomilin [22] except for the lack of a furan ring and the presence of a β-substituted γ-hydroxybutenolide group. The following correlations were also observed in the ^1^H-^1^H COSY experiment; H-1/H_2_-2; H-5/H_2_-6; H-9/H_2_-11; H_2_-11/H_2_-12. In the NOESY experiment, the following correlations were observed; Me-28/H-6α; Me-18/H-9α, H-22, and 1-OCOCH_3_; H-2β/Me-19 and Me-29; H-6β/Me-19 and Me-30; H-12β/H-17 (Figure 3). Therefore, the structure of **1** was established, as shown in Figure 1. Compound **1** was an inseparable mixture of C-21 hemiacetal epimers.

21,23-dihydro-23-methoxy-21-oxonomilin (**2**) was obtained as colorless crystals. Its molecular formula was established as C_29_H_32_O_11_ (*m*/*z* 561.2346 [M + H]^+^, calcd.: 561.2336) by the HRFABMS. Compound **2** was suggested to have a similar structure to **1** except for the absence of a β-substituted γ-hydroxybutenolide group and the presence of an α-substituted γ-methoxybutenolide group because of the HMBC correlations from H-17 (δ_H_ 5.32 (t)) to an ester carbonyl carbon (δ_C_ 169.8 (s)) and a trisubstituted olefin (δ_C_ 134.0 (s), 150.9 (d)). Therefore, the structure of **2** was established, as shown in Figure 1. The configuration at C-23 was not determined.

21,23-dihydro-21-hydroxy-23-oxonomilinic acid methyl ester (**3**), an amorphous solid, possessed the molecular formula C_29_H_38_O_12_ (*m*/*z* 601.2264 [M + Na]^+^, calcd.: 601.2261). The ^1^H- and ^13^C-NMR spectra showed three methyls observed as singlet including acetyl and methoxy groups (δ_H_ 1.10 (s), 1.14 (s), 1.31 (s), 2.08 (s), 3.67 (s)), a hydroxy propyl (δ_H_ 1.34 (s), 1.35 (s); δ_C_ 74.1 (s)), three oxymethine (δ_H_ 3.64 (s), 5.33 (d), 6.54 (brs); δ_C_ 52.5 (d), 76.3 (d), 78.6 (d)), a hemiacetal (δ_H_ 6.00 (brs); δ_C_ 98.2 (d)), an unprotonated oxycarbon (δ_C_ 65.0 (s)), a trisubstituted olefin (δ_H_ 6.29 (brs); δ_C_ 122.7 (d), 163.6 (s)), a ketone (δ_C_ 210.0 (s)), four ester carbonyls (δ_C_ 166.3 (s), 169.8 (s), 170.8 (s), 172.0 (s)). In the HMBC experiment, the following correlations were observed: Me-18 (δ_H_ 1.10 (s))/C-12, C-13, C-14 (δ_C_ 65.0 (s)), C-17 (δ_C_ 78.6 (d)); Me-19 (δ_H_ 1.31 (s))/C-1 (δ_C_ 76.3 (d)), C-5, C-9, C-10; Me-28 (δ_H_ 1.35 (s)) and Me-29 (δ_H_ 1.34 (s))/C-4 (δ_C_ 74.1 (s)), C-5; Me-30 (δ_H_ 1.14 (s))/C-7 (δ_C_ 210.0 (s)), C-8, C-9, C-14; H-2B (δ_H_ 2.82 (m)), 3-OCH_3_ (δ_H_ 3.67 (s))/C-3 (δ_C_ 172.0 (s)); H-15 (δ_H_ 3.64 (s))/C-16 (δ_C_ 166.3 (s)); H-17 (δ_H_ 5.33 (s))/C-20 (δ_C_ 163.6 (s)), C-22 (δ_C_ 122.7 (d)); H-22 (δ_H_ 6.29 (brs))/C-21 (δ_C_ 98.2 (d)), C-23 (δ_C_ 169.8 (s)). The following correlations were also observed in the ^1^H-^1^H COSY experiment: H-1 (δ_H_ 6.54 (brs))–H-2A; H-5–H_2_-6; H-9–H_2_-11–H_2_-12. From the above data, compound **3** was similar to 3-*O*-methyl 21,23-dihydro-23-hydroxy-21-oxonomilinic acid [23] except for the absence of an α-substituted γ-hydroxybutenolide group and the presence of a β-substituted γ-hydroxybutenolide group. The relative configuration was determined by the NOESY experiment (Figure 3). The configuration of C-1 was determined as *S* because of the following NOE correlations: H-1 (δ_H_ 6.54 (brs))/H-2B (δ_H_ 2.82 (m)), Me-19 (δ_H_ 1.31 (s)), Me-28 (δ_H_ 1.35 (s)), and Me-29 (δ_H_ 1.34 (s)); H-2A (δ_H_ 2.35 (m))/H-9 (δ_H_ 2.14 (brd)); Me-19/Me-2′ (δ_H_ 3.67 (s)). Therefore, the structure of **3** was established as shown in Figure 1. Compound **3** was also an inseparable mixture of C-21 hemiacetal epimers.

**Figure 4:**
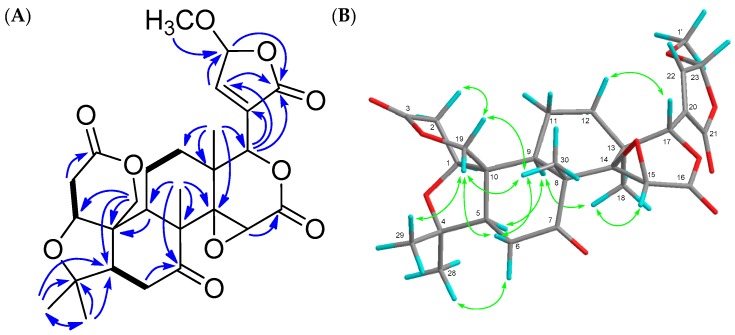
Key HMBC (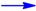) and ^1^H-^1^H COSY (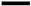) (**A**) and NOE (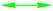) (**B**) correlations of compound **4**.

21,23-dihydro-23-methoxy-21-oxolimonin (**4**) was obtained as an amorphous solid. Its molecular formula was established as C_27_H_32_O_10_ (*m*/*z* 539.1891 [M + Na]^+^, calcd.: 539.1894) by the HRFABMS. The absorbance in the IR spectrum indicated hydroxy (3434 cm^−1^) and carbonyl (1750 cm^−1^) groups. The ^1^H- and ^13^C-NMR spectra indicated five methyl groups observed as singlets including methoxy group (δ_H_ 1.09 (s), 1.18 (s), 1.18 (s), 1.29 (s), 3.60 (s)), an oxymethylene (δ_H_ 4.46 (d), 4.74 (d); δ_C_ 65.1 (t)) three oxymethines (δ_H_ 4.03 (brd), 4.12 (s), 5.43 (t); δ_C_ 53.8 (d), 75.2 (d), 79.2 (d)), two unprotonated oxycarbons (δ_C_ 65.7 (s), 80.3 (s)), an acetal (δ_H_ 5.77 (t); δ_C_ 102.5 (d)) a trisubstituted olefin (δ_H_ 7.25 (t); δ_C_ 133.8 (s), 149.1 (d)), a ketone (δ_C_ 206.1 (s)), and three ester carbonyls (δ_C_ 166.0 (s), 168.8 (s), 168.9 (s)) (Table 2). The HMBC and ^1^H-^1^H COSY correlations (Figure 4) suggested that compound **4** had a similar structure to limonin [15] except for the lack of a furan ring and presence of an α-substituted γ-methoxybutenolide group. In the NOESY experiment, the following correlations were observed; Me-29/H-19β; H-19β/H-6β, Me-30; H-12β/H-17; H-6α/Me-28; H-5/H-9; H-9/Me-18; Me-18/23-OMe; Me-30/H-19α; H-19α/H-2α (Figure 4). Therefore, the structure of **4** was established, as shown in Figure 1. The configuration at C-23 was not established.

21,23-Dihydro-21-oxolimonin (**5**) was obtained as an amorphous solid. Its molecular formula was established as C_26_H_30_O_9_ (*m*/*z* 487.1967 [M + H]^+^, calcd.: 487.1968). Compound **5** was similar to **4** except for the absence of an acetal carbon and the presence of an oxymethylene at C-23. The structure was confirmed by 2D NMR spectra including HSQC, HMBC, ^1^H-^1^H COSY, and NOESY experiments. Therefore, the structure of **5** was established, as shown in Figure 1.

Macrophages may be a potential therapeutic target for inflammatory diseases [24]. Activated macrophages release pro-inflammatory mediators, such as NO, reactive oxygen species, interleukin-1β, tumor necrosis factor-α, and other inflammatory mediators, which play important roles in biological defense. However, the overexpression of these mediators had been implicated in diseases such as osteoarthritis, rheumatoid arthritis, and diabetes because the increased production of pro-inflammatory mediators induces severe or chronic inflammation [24]. Isolated compounds (**1**–**12**), and *N*^G^-monomethyl-l-arginine, acetate (l-NMMA), which is a NO synthase inhibitor and was used as a positive control, were evaluated for macrophage activation by the inhibitory assay of NO production in RAW264.7 mouse macrophages stimulated by lipopolysaccharide (LPS). Compounds **1**–**7**, and **9**–**12** did not exhibit cytotoxicity at 1–30 μM (Table 3). Of these, compound **2** (IC_50_ 25.4 μM) showed a comparable inhibitory effect on NO production to l-NMMA (IC_50_ 23.9 μM) (Table 3). Compounds **3**, **5**, **8**, **11** and **12** showed some inhibitory activities (produced NO ratio **3**: 80.3%; **5**: 83.2%; **8**: 76.0%; **11**: 78.1%; **12**: 73.9% at 30 μM). The other limonoids did not exhibit inhibitory effects on NO production. These results suggested that compound **2** has potential as an anti-inflammatory disease agent.

## 3. Experimental

### 3.1. General Experimental Procedure

The following chemicals and reagents were purchased: fetal bovine serum (FBS) from Invitrogen Co. (Carlsbad, CA, USA), 3-(4,5-dimethyl-2-thiazolyl)-2,5-diphenyl-2*H*-tetrazolium bromide (MTT) from Sigma-Aldrich Japan Co. (Tokyo, Japan), Dulbecco’s modified Eagle’s medium (D-MEM), and antibiotics from Nacalai Tesque, Inc. (Kyoto, Japan). All other chemicals and reagents were of analytical grade. Melting points were determined on a Yanagimoto micro-melting point apparatus and were uncorrected. Optical rotations were measured with a JASCO DIP-1000 digital polarimeter. IR spectra were recorded on a Perkin-Elmer 1720X FTIR spectrophotometer. UV spectra were measured on a HITACHI U-2000 spectrometer. ^1^H- (600 MHz) and ^13^C- (150 MHz) NMR spectra were recorded on an Agilent vnmrs600 with tetramethylsilane as the internal standard. HR-FAB-MS was recorded on a JEOL JMS-7000 mass spectrometer. Silica gel (70–230 mesh, Merck) and silica gel 60 (230–400 mesh, Nacalai tesque, Inc., Kyoto, Japan) were used for column chromatography and medium-pressure liquid chromatography, respectively. HPLC was carried out on an ODS column (*Cosmosil 5C_18_-MS-II column* (Nacalai Tesque, Inc., Kyoto, Japan), 25 cm × 20 mm i.d., 5 μm particle size) at 35 °C with MeCN/H_2_O (1:1 (System *I*), 45:55 (System *III*), 2:3 (System *IV*), 3:7 (System *V*), flow rate 4.0 mL/min), *Cosmosil PAQ* (Nacalai Tesque, Inc., Kyoto, Japan), 25 cm × 20 mm i.d., 5 μm particle size) at 35 °C (MeCN/H_2_O, flow rate 4.0 mL/min, (1:1) (System *II*)).

### 3.2. Plant Material

The fruits of *C. reticulata* were produced in Wakayama prefecture (Japan) in 2013. A voucher specimen of the peels of *Citrus reticulata* was deposited in the Herbarium of the Laboratory of Medicinal Chemistry, Osaka University of Pharmaceutical Sciences.

### 3.3. Extraction and Isolation

The peels of *Citrus reticulata* fruits (dry weight 4952 g), produced in Wakayama, were subjected to extraction with MeOH under reflux (15 L, 3 days, 3 times). The MeOH extract (280 g) was then partitioned between AcOEt and H_2_O (9 L/9 L, 4 times). The AcOEt-soluble fraction (280 g) was subjected to SiO_2_ column chromatography (CC) (SiO_2_ (3.5 kg); hexane/AcOEt (5:1, 1:1, and 0:1), and AcOEt:MeOH (1:1, and 0:1) in increasing order of polarity) resulting in twenty fractions (Fr. A–T).

Fr. J (11 g), eluted with AcOEt, was subjected to SiO_2_ CC to yield 10 fractions (SiO_2_ (300 g); hexane/AcOEt (5:1, 3:1, 1:1, 1:5, 1:10, 1:20, and 0:1), and AcOEt:MeOH (5:1, and 0:1) in increasing order of polarity), J1–J10. Preparative HPLC (System *III*) of J6 (237 mg), eluted with hexane/AcOEt (1:10), gave **5** (4.5 mg; t_R_ 24.6 min).

Fr. K (11 g), eluted with AcOEt, was subjected to SiO_2_ CC to yield 11 fractions (SiO_2_ (270 g); hexane/AcOEt (1:1 and 0:1), and AcOEt:MeOH (1:1, and 0:1) in increasing order of polarity), K1–K11, followed by SiO_2_ CC of K5 (1 g), eluted with AcOEt, to yield 8 fractions (SiO_2_ (60 g); AcOEt and MeOH), K5-1–K5-8. Preparative HPLC (System *IV*) of K5-2 (215 mg), eluted with AcOEt, gave 12 fractions, K5-2-1–K5-2-12. K5-2-9 was identified as **6** (26 mg; t_R_ 47.4 min). Preparative HPLC (System *V*) of K5-2-2 (11 mg) gave **7** (5.4 mg; t_R_ 47.7 min). Preparative HPLC (System *V*) of K5-2-3 (20 mg) gave **8** (3.0 mg; t_R_ 58.6 min) and **9** (1.3 mg; t_R_ 49.0 min). Preparative HPLC (System *V*) of K5-2-6 (10 mg) gave **10** (1.9 mg; t_R_ 91.8 min). Preparative HPLC (System *V*) of K5-2-8 (7.9 mg) gave **3** (1.1 mg; t_R_ 92.0 min).

Preparative HPLC (System *IV*) of K5-3 (397 mg) gave **8** (3.7 mg; t_R_ 58.8 min). Fr. K6 (3 g), eluted with AcOEt, was subjected to SiO_2_ CC, to yield 8 fractions (SiO_2_ (60 g); AcOEt and MeOH), K6-1–K6-8. Preparative HPLC (System *I*) of K6-2 (376 mg), eluted with AcOEt, gave 10 fractions, K6-2-1–K6-2-10. Preparative HPLC (System *V*) of K6-2-1 (135 mg) gave **1** (14 mg; t_R_ 104.5 min) and **2** (2.4 mg; t_R_ 186.8 min). Preparative HPLC (System *IV*) of K6-2-3 (4.2 mg) gave **4** (2.5 mg; t_R_ 51.6 min).Preparative HPLC (System *II*) of K6-2-3 (1120 mg), eluted with AcOEt, gave 15 fractions, K6-3-1–K6-3-15. K6-3-9 was identified as **11** (42 mg; t_R_ 27.8 min). Preparative HPLC (System *V*) of K6-3-4 (59 mg) gave **1** (12 mg) and **12** (5.5 mg; t_R_ 92.6 min).

*21,23-Dihydro-21-hydroxy-23-oxonomilin* (**1**). Colorless crystals, m.p. 200–201 °C; ${\left[\alpha \right]}_{D}^{21}$ −100.0 (*c* = 0.083, EtOH); IR ν_max_^KBr^ cm^−1^: 3466, 2921, 2852, 1747, 1733, 1717, 1596, 1378, 1232, 1025; UV λ_max_^EtOH^ nm (logε): 211.5 (3.82); FAB-MS *m*/*z*: 547 [M + H]^+^, 569 [M + Na]^+^; HR-FAB-MS *m*/*z*: 547.2178 (calcd. for 547.2179: C_28_H_35_O_11_).

*21,23-Dihydro-23-methoxy-21-oxonomilin* (**2**). Colorless crystals, m.p. 208–210 °C; ${\left[\alpha \right]}_{D}^{12}$ −15.7 (*c* = 0.075, EtOH); IR ν_max_^KBr^ cm^−1^: 3425, 2925, 1751, 1716, 1374, 1120, 1027; UV λ_max_^EtOH^ nm (logε): 212.5 (3.72); FAB-MS *m*/*z*: 561 [M + H]^+^, 583 [M + Na]^+^; HR-FAB-MS *m*/*z*: 561.2346 (calcd. for 561.2336: C_29_H_37_O_11_).

*21,23-Dihydro-21-hydroxy-23-oxonomilinic acid methyl ester* (**3**). Amorphous solid; ${\left[\alpha \right]}_{D}^{25}$ −54.7 (*c* = 0.16, EtOH); IR ν_max_^KBr^ cm^−1^: 3453, 2924, 2853, 1743, 1596, 1442, 1382, 1261, 1028; UV λ_max_^EtOH^ nm (logε): 203.5 (3.80); FAB-MS *m*/*z*: 601 [M + Na]^+^; HR-FAB-MS *m*/*z*: 601.2264 (calcd. for 601.2261: C_29_H_38_NaO_12_).

*21,23-Dihydro-23-methoxy-21-oxolimonin* (**4**). Amorphous solid; ${\left[\alpha \right]}_{D}^{23}$ −15.7 (*c* = 0.12, EtOH); IR ν_max_^KBr^ cm^−1^: 3434, 2922, 2852, 1750, 1713, 1632, 1456, 1384, 1034; UV λ_max_^EtOH^ nm (logε): 206.5 (3.43); FAB-MS *m*/*z*: 539 [M + Na]^+^; HR-FAB-MS *m*/*z*: 539.1891 (calcd. for 539.1894: C_27_H_32_NaO_10_).

*21,23-Dihydro-21-oxolimonin* (**5**)*.* Amorphous solid; ${\left[\alpha \right]}_{D}^{25}$ −53.9 (*c* = 0.10, EtOH); IR ν_max_^KBr^ cm^−1^: 2969, 1749, 1712, 1457, 1361, 1265, 1095; UV ν_max_^EtOH^ nm (logε): 206.0 (3.76); FAB-MS *m*/*z*: 487 [M + H]^+^; HR-FAB-MS *m*/*z*: 487.1967 (calcd. for 487.1968: C_26_H_31_O_9_).

### 3.4. Cell Culture

RAW264.7 cells were grown in D-MEM medium supplemented with 10% FBS and antibiotics (100 units/mL penicillin G sodium salt and 100 μg/mL streptomycin sulfate). Cells were incubated at 37 °C in a 5% CO_2_ humidified incubator.

### 3.5. Cytotoxicity Assay

Cytotoxicity assay was performed according to a method reported previously [25] Briefly, RAW264.7 cells (5 × 10^4^ cells in 100 μL) were seeded onto a 96-well microplate and incubated for 24 h. DMEM containing test samples (100 μL total volume, final concentration of 30, 10, 3, or 1 μM) dissolved in DMSO (final concentration 0.2%) was added. After treatment for 24 h, MTT solution was added. After a 3-h incubation, 20% sodium dodecyl sulfate in 0.1 M HCl was added to dissolve the formazan produced in the cells. The absorbance of each well was read at 570 nm using a microplate reader. The optical density of vehicle control cells was assumed to be 100%.

### 3.6. Inhibitory Assay of NO Production

An inhibitory assay of NO production was performed according to a method reported previously [26] with slight modifications. Briefly, RAW264.7 cells (5 × 10^4^ cells in 100 μL) were seeded into a 96-well microplate and incubated for 24 h. DMEM containing test samples (100 μL total volume, final concentration of 30, 10, 3, or 1 μM) dissolved in DMSO (final concentration 0.2%), and LPS (final concentration of 5 μg/mL), was added. After treatment for 24 h, the supernatant of culture medium was transferred to another 96-well microplate, and then 50 μL of 0.15% *N*-(1-naphtyl)ethylenediamine in H_2_O, and 1.5% sulfanilamide in 7.5% phosphoric acid were added. After incubation for 30 min, the absorbance of each well was read at 570 nm using a microplate reader. The optical density of vehicle control cells was assumed to be 100%.

## 4. Conclusions

In this study, we isolated five new compounds and elucidated their structures. They were limonoids having β-substituted γ-hydroxybutenolide (**1**, **3**), α-substituted γ-methoxybutenolide (**2**, **4**), and α-substituted butenolide (**5**) groups. Compounds **1** and **3** were inseparable mixtures of C-21 hemiacetal epimers. In NO inhibitory assay, compound **2** (IC_50_ 25.4 μM) possessed a comparable inhibitory effect on NO production to l-NMMA (IC_50_ 23.9 μM) without cytotoxicities. These results suggested that compound **2** has potential as an anti-inflammatory disease agent.

## Figures and Tables

**Table 1:** ^1^H- (600 MHz) and ^13^C-NMR (150 MHz) spectra data of compounds **1** (in CDCl_3_), **2** (in (CD_3_)_2_CO) and **3** (in CDCl_3_ + 1 drop CD_3_OD) ^a^.

Position	1				2				3			
	δ_H_	Mult. (*J* in Hz)	δ_C_		δ_H_	Mult. (*J* in Hz)	δ_C_		δ_H_	Mult. (*J* in Hz)	δ_C_	
1β	5.05	brd (6.4)	70.7	d	4.97	d (7.1)	72.0	d	6.54	brs	76.3	d
2	α 3.11	dd (6.4, 15.8)	35.3	t	α 2.98	dd (7.1, 15.8)	36.0	t	A 2.35	m	35.4	t
	β 3.24	brd (15.8)			β 3.50	dd (1.2, 15.8)			B 2.82	m		
3			169.1	s			169.5	s			172.0	s
4			84.4	s			84.7	s			74.1	s
5	2.62	dd (3.9, 15.8)	51.1	d	2.69	dd (3.5, 14.7)	51.7	d	2.01	m	53.0	
6α	α 2.60	dd (3.9, 15.8)	38.6	t	α 2.51	dd (3.5, 14.7)	39.6	t	α 2.46	dd (5.3, 14.9)	38.9	t
6β	β 2.78	t (15.8)			β 3.10	t (14.7)			β 2.80	t (14.9)		
7			206.5	s			208.3	s			210.0	s
8			53.1	s			53.2	s			52.5	s
9	2.44	brd (11.2)	44.3	d	2.61	dd (2.4, 11.8)	44.8	d	2.14	brd (11.4)	44.4	d
10			44.1	s			45.1	s			46.1	s
11	α 1.70	m	17.3	t	α 1.63	m	17.44	t	α 2.49	m	19.0	t
	β 1.66	m			β 1.73	m			β 1.72	m		
12	α 1.29	m	32.0	t	α 1.16	m	30.6	t	α 1.61	m	31.6	t
	β 2.02	dd (7.9, 13.0)			β 2.10	m			β 2.01	m		
13			37.7	s			39.0	s			37.5	s
14			65.0	s			66.8	s			65.0	s
15	3.70	s	52.6	d	3.95	m	54.5	d	3.64	s	52.5	d
16			165.5	s			166.9				166.3	s
17	5.34	brd (1.7)	78.1	d	5.32	t (1.1)	76.2	d	5.33	d (1.5)	78.6	d
18	1.11	s	21.4	q	1.20	s	20.4	q	1.10	s	21.4	q
19	1.34	s	16.64	q	1.47	m	16.1	q	1.31	s	16.7	q
20			162.5	s			134.0	s			163.6	s
21	5.99	brs	97.4	d			169.80	s	6.00	brs	98.2	d
22	6.30	dd (0.9, 1.7)	123.2	d	7.48	t (1.1)	150.9	d	6.29	brs	122.7	d
23			168.9	s	6.02	t (1.1)	103.5	d			169.8	s
28	1.48	s	33.4	q	1.39	s	33.9	q	1.35	s	33.6	q
29	1.56	s	23.4	q	1.64	s	23.3	q	1.34	s	27.5	q
30	1.16	s	16.66	q	1.28	s	17.49	q	1.14	s	16.3	q
1′			169.6	s	2.00	s	20.7	q			170.8	s
2′	2.10	s	20.9	q			169.82	s	2.08	s	21.1	q
3-OCH_3_									3.67	s	52.3	q
23-OCH_3_					3.45	s	56.9	q				

^a^ Assignments were based on ^1^H-^1^H COSY, HMQC, HMBC, and NOESY spectroscopic data.

**Table 2:** ^1^H- (600 MHz) and ^13^C-NMR (150 MHz) spectra data of compounds **4** (in CDCl_3_) and **5** (in (CD_3_)_2_CO) ^a^.

Position	4				5			
	δ_H_	Mult. (*J* in Hz)	δ_C_		δ_H_	Mult. (*J* in Hz)	δ_C_	
1β	4.03	brd (4.2)	79.2	d	4.27	brd (4.1)	80.1	d
2α	2.66	dd (1.7, 16.8)	35.6	t	2.87	dd (1.5, 16.7)	36.5	t
2β	2.98	dd (4.2, 16.8)			2.73	dd (4.1, 16.7)		
3			168.9	s			170.0	s
4			80.3	s			80.7	s
5	2.22	m	60.4	d	2.60	dd (3.6, 15.2)	59.8	d
6α	2.47	dd (3.5, 14.6)	36.3	t	2.40	dd (3.6, 15.2)	37.1	t
6β	2.84	dd (14.6, 15.8)			3.16	t (15.2)		
7			206.1	s			208.2	s
8			51.1	s			51.7	s
9	2.50	dd (3.3, 12.7)	48.0	d	2.83	m	48.2	d
10			45.8	s			46.7	s
11α	1.77	(2H)	18.5	t	1.96	m	18.8	t
11β					2.07	m		
12α	1.40	ddd (7.3, 9.1, 14.4)	28.7	t	1.44	m	29.0	t
12β	2.24	m			2.06	m		
13			38.6	s			39.9	s
14			65.7	s			67.6	s
15	4.12	s	53.8	d	4.19	s	55.2	d
16			166.0	s			167.3	s
17	5.43	t (1.5)	75.2	d	5.35	d (1.2)	76.6	d
18	1.18	s	20.0	q	1.27	s	19.7	q
19α	4.46	d (13.2)	65.1	t	4.65	d (13.5)	65.7	t
19β	4.74	d (13.2)			4.97	d (13.5)		
20			133.8	s			129.6	s
21			168.8	s			173.1	s
22	7.25	t (1.5)	149.1	d	7.85	dd (1.7, 2.9)	154.4	d
23	5.77	t (1.5)	102.5	d	4.99	dd (1.7, 3.5)	71.9	t
28	1.29	s	30.2	q	1.13	s	21.8	q
29	1.18	s	21.2	q	1.24	s	30.3	q
30	1.09	s	17.8	q	1.18	s	18.2	q
23-OCH_3_	3.60	s	57.8	q				

^a^ Assignments were based on ^1^H-^1^H COSY, HMQC, HMBC, and NOESY spectroscopic data.

**Table 3:** Inhibitory effects of NO production by limonoids from peels of *Citrus reticulata.*

Inhibitory Ratio of NO % (Cell Viability %) ^a,b^					
Compound	1 μM	3 μM	10 μM	30 μM	IC_50_ (μM)
**1**	109.9 ± 2.8	110.9 ± 1.7	104.8 ± 2.2	106.6 ± 2.5	>30
	(99.1 ± 1.3)	(102.5 ± 1.8)	(103.7 ± 0.7)	(99.3 ± 1.3)	
**2**	95.2 ± 1.6	89.8 ± 2.1 *	80.4 ± 2.1 **	39.3 ± 0.5 **	25.4
	(101.3 ± 1.5)	(98.7 ± 1.4)	(99.1 ± 0.3)	(94.5 ± 0.4)	
**3**	99.4 ± 0.8	99.4 ± 2.3	94.1 ± 1.9	80.3 ± 1.2 **	>30
	(96.8 ± 0.2)	(94.5 ± 0.3)	(93.2 ± 0.4)	(93.4 ± 0.6)	
**4**	96.2 ± 7.9	94.0 ± 2.7	100.8 ± 1.2	95.2 ± 3.4	>30
	(102.6 ± 0.4)	(97.0 ± 0.3)	(95.1 ± 0.2)	(91.7 ± 0.5)	
**5**	99.5 ± 5.6	100.6 ± 4.7	93.8 ± 1.6	83.2 ± 5.6*	>30
	(97.9 ± 1.9)	(97.9 ± 0.2)	(99.5 ± 0.5)	(96.6 ± 0.2)	
**6**	102.3 ± 4.4	93.4 ± 5.7	90.9 ± 3.1	86.0 ± 5.5	>30
	(100.1 ± 0.3)	(95.6 ± 0.5)	(97.2 ± 0.9)	(99.3 ± 0.9)	
**7**	99.8 ± 1.4	99.0 ± 3.0	96.8 ± 2.5	93.9 ± 4.0	>30
	(102.3 ± 1.5)	(106.3 ± 2.2)	(104.2 ± 1.4)	(100.2 ± 1.4)	
**8**	97.2 ± 1.1	94.7 ± 2.4	90.9 ± 1.6 **	76.0 ± 2.2 **	>30
	(98.1 ± 2.4)	(97.6 ± 0.7)	(92.0 ± 0.5)	(89.3 ± 0.5)	
**9**	94.3 ± 4.3	85.2 ± 6.4	91.3 ± 2.8	89.9 ± 3.2	>30
	(101.4 ± 0.7)	(97.7 ± 0.6)	(101.3 ± 1.2)	(97.6 ± 2.1)	
**10**	95.0 ± 4.2	96.8 ± 1.3	97.7 ± 2.2	86.6 ± 1.6	>30
	(104.7 ± 2.3)	(101.8 ± 1.0)	(98.9 ± 0.5)	(98.6 ± 1.8)	
**11**	103.6 ± 3.1	93.2 ± 3.2	84.7 ± 3.2 *	78.1 ± 3.4 **	>30
	(95.9 ± 0.1)	(100.1 ± 0.4)	(99.3 ± 1.5)	(100.4 ± 0.6)	
**12**	106.6 ± 4.1	92.6 ± 3.2	88.4 ± 5.5	73.9 ± 3.4 **	>30
	(100.7 ± 0.3)	(98.1 ± 1.0)	(98.3 ± 1.6)	(98.3 ± 1.2)	
**l-NMMA ^c^**	93.3 ± 2.2	91.4 ± 0.8	68.9 ± 4.5 **	43.1 ± 1.1 **	23.9
	(101.5 ± 0.9)	(101.9 ± 0.4)	(98.5 ± 0.9)	(109.4 ± 0.5)	

^a^ Each value represents the mean ± standard error (S.E.) of four determinations; ^b^ Significant differences from the vehicle control group shown as * *p* < 0.05 and ** *p* < 0.01; ^c^ Positive control.

## Supplementary Materials

Supplementary materials are available online.
