# Supplementary material for: Five New Limonoids from Peels of Satsuma Orange (Citrus reticulata)
Source: Molecules. 2017 May 31;22(6):907. doi: 10.3390/molecules22060907 (PMC6152666; doi:10.3390/molecules22060907)

Supplementary Data

Takashi Kikuchi [1], Yasuaki Ueno [1], Yoshino Hamada [1], Chika Furukawa [1], Takako Fujimoto [1], Takeshi Yamada [1], and Reiko Tanaka [1,*]

[1] Osaka University of Pharmaceutical Sciences, 4-20-1 Nasahara, Takatsuki, Osaka 569-1142, Japan;
* Correspondence: tanakar@gly.oups.ac.jp; Tel.: +81-72-690-1084

Content

Table S1. NMR Data of **1**.······1
Table S2. NMR Data of **2**.······2
Table S3. NMR Data of **3**.······3
Table S4. NMR Data of **4**.······4
Table S5. NMR Data of **5**.······5
Fig. S1. ^{1}H-NMR spectrum of compound **1**.······6
Fig. S2. ^{13}C-NMR spectrum of compound **1**.······7
Fig. S3. HSQC spectrum of compound **1**.······8
Fig. S4. HMBC spectrum of compound **1**.······9
Fig. S5. ^{1}H-^{1}H COSY spectrum of compound **1**.······10
Fig. S6. NOESY spectrum of compound **1**.······11
Fig. S7. ^{1}H-NMR spectrum of compound **2**.······12
Fig. S8. ^{13}C-NMR spectrum of compound **2**.······13
Fig. S9. HSQC spectrum of compound **2**.······14
Fig. S10. HMBC spectrum of compound **2**.······15
Fig. S11. ^{1}H-^{1}H COSY spectrum of compound **2**.······16
Fig. S12. NOESY spectrum of compound **2**.······17
Fig. S13. ^{1}H-NMR spectrum of compound **3**.······18
Fig. S14. ^{13}C-NMR spectrum of compound **3**.······19
Fig. S15. HSQC spectrum of compound **3**.······20
Fig. S16. HMBC spectrum of compound **3**.······21
Fig. S17. ^{1}H-^{1}H COSY spectrum of compound **3**.······22
Fig. S18. NOESY spectrum of compound **3**.······23
Fig. S19. ^{1}H-NMR spectrum of compound **4**.······24
Fig. S20. ^{13}C-NMR spectrum of compound **4**.······25
Fig. S21. HSQC spectrum of compound **4**.······26
Fig. S22. HMBC spectrum of compound **4**.······27
Fig. S23. ^{1}H-^{1}H COSY spectrum of compound **4**.······28
Fig. S24. NOESY spectrum of compound **4**.······29
Fig. S25. ^{1}H-NMR spectrum of compound **5**.······30
Fig. S26. ^{13}C-NMR spectrum of compound **5**.······31
Fig. S27. HSQC spectrum of compound **5**.······32
Fig. S28. HMBC spectrum of compound **5**.······33
Fig. S29. ^{1}H-^{1}H COSY spectrum of compound **5**.······34
Fig. S30. NOESY spectrum of compound **5**.······35

Table S1. NMR Spectral Data of **1** in $CDCl_3$.

No.	δ_H[a]	(J in Hz)	^1H-^1H COSY	NOE	δ_C		HMBC (C)[b]
1β	5.05	brd (6.4)	2α, 2β		70.7	d	2, 3, 5, 9, 30, 1'
2α	3.11	dd (6.4, 15.8)	1, 2β	19, 29	35.3	t	1, 3, 10
2β	3.24	brd (15.8)	1, 2α				1, 3, 10
3					169.1	s	
4					84.4	s	
5	2.62	dd (3.9, 15.8)	6α, 6β	9	51.1	d	19, 29, 30
6α	2.60	dd (3.9, 15.8)	5, 6β		38.6	t	4, 7, 8, 10
6β	2.78	t (15.8)	5, 6α	19, 29, 30			4, 5, 7, 10
7					206.5	s	
8					53.1	s	
9	2.44	d	11α, 11β		44.3	d	5, 7, 8, 10, 12, 30
10					44.1	s	
11α	1.70	m	9, 11β, 12α, 12β		17.3	t	8, 9
11β	1.66	m	9, 11α, 12α, 12β	30			13
12α	1.29	m	11α, 11β, 12β		32.0	t	13, 14, 17
12β	2.02	dd (7.9, 13.0)	11α, 11β, 12α	17, 21			9, 11, 13, 14, 17, 18
13					37.7	s	
14					65.0	s	
15	3.70	brs		18	52.6	d	14, 16
16					165.5	s	
17	5.34	brs		12β, 21	78.1	d	12, 13, 14, 18, 20, 21, 22
18	1.11	s		2'	21.4	q	12, 13, 14, 17
19	1.34	s		2β, 6β	16.64	q	1, 5, 9, 10
20					162.5	s	
21	5.99	m		12β, 17	97.4	d	
22	6.30	m			123.2	d	17, 21, 23
23					168.9	s	
28	1.48	s		6α	33.4	q	4, 5, 29
29	1.56	s		2β, 6β	23.4	q	4, 5, 28
30	1.16	s		6β, 11β	16.66	q	7, 8, 9, 14
1'					169.6	s	
2'	2.10	s		18	20.9	q	1'

[a] ^1H chemical shift values (δ ppm from $SiMe_4$) followed by multiplicity and then the coupling constants (J/Hz).

[b] Long range ^1H-^{13}C correlation from H to C observed in the HMBC experiment.

Table S2. NMR Spectral Data of **2** in $(CD_3)_2CO$.

No.	δ_H[a]	(J in Hz)	^{1}H-^{1}H COSY	NOE	δ_C		HMBC (C)[b]
1	4.97	d (7.1)	2α, 2β		72.0	d	2, 3, 5, 10, 19, 1'
2α	2.98	dd (7.1, 15.8)	1, 2β		36.0	t	3, 10
2β	3.50	dd (1.2, 15.8)	1, 2α	29			3, 10
3					169.5	s	
4					84.7	s	
5	2.69	dd (3.5, 14.7)	6α, 6β	9	51.7	d	4, 6, 10, 19, 29
6α	2.51	dd (3.5, 14.7)	5, 6β		39.6	t	5, 7, 8, 10
6β	3.10	t (14.7)	5, 6α	19			4, 5, 7, 10
7					208.3	s	
8					53.2	s	
9	2.61	dd (2.4, 11.8)	11α, 11β	5, 18	44.8	d	5, 8, 10, 12, 19, 30
10					45.1	s	
11α	1.63	m	9, 12α, 12β		17.44	t	
11β	1.73	m	9, 12α, 12β	19, 30			
12α	1.16	m	11α, 11β, 12β		30.6	t	18, 30
12β	2.10	m	11α, 12α	17, 30			
13					39.0	s	
14					66.8	s	
15	3.95	s		18	54.5	d	16
16					166.9	s	
17	5.32	t (1.1)	22, 23	12α	76.2	d	13, 14, 18, 20, 21, 22
18	1.20	s		9, 19	20.4	q	12, 13, 14, 17
19	1.47	s		18	16.1	q	1, 5, 9, 10
20					134.0	s	
21					169.80	s	
22	7.48	t (1.1)	17, 23		150.9	d	17, 20, 21, 23
23	6.02	t (1.1)	17, 22		103.5	d	20, 21, 23-O<u>CH$_3$</u>
28	1.39	s			33.9	q	4, 5, 29
29	1.64	s		2β, 19	23.3	q	4, 5, 28
30	1.28	s		11β, 12β	17.49	q	7, 8, 9, 14
1'					169.82	s	
2'	2.00	s			20.7	q	1'
23-O<u>CH$_3$</u>	3.45	s			56.9	q	23

[a] ^{1}H chemical shift values (δ ppm from $SiMe_4$) followed by multiplicity and then the coupling constants (J/Hz).

[b] Long range ^{1}H-^{13}C correlation from H to C observed in the HMBC experiment.

Table 3S. NMR Spectral Data of **3** in $CDCl_3$+1 drop CD_3OD.

No.		δ_H[a] (J in Hz)		^1H-^1H COSY	NOE	δ_C		HMBC (C)[b]
1		6.54	brs	2A	2B, 19, 28, 29	76.3	d	
2	A	2.35	m	1, 2B	9	35.4	t	
	B	2.82	m	2A	1, 5, 9			3
3						172.0	s	
4						74.1	s	
5		2.01	m	6α, 6β	2B, 9	53.0		
6	α	2.46	dd (5.3, 14.9)	6β		38.9	t	5, 7, 8
	β	2.80	t (14.9)	6α	19			4, 5, 7, 8
7						210.0	s	
8						52.5	s	
9		2.14	d (11.4)	11α, 11β	2A, 2B, 18	44.4	d	5, 7, 8, 12, 30
10						46.1	s	
11	α	2.49	m	9, 11β, 12α, 12β		19.0	t	
	β	1.72	m	9, 11α, 12α, 12β	19, 30			8, 12
12	α	1.61	m	11α, 12β		31.6	t	14
	β	2.01	m	11α, 12α	17			11, 17
13						37.5	s	
14						65.0	s	
15		3.64	s			52.5	d	14, 16
16						166.3	s	
17		5.33	d (1.5)	22	12β	78.6	d	14, 20, 22
18		1.10	s		9, 21, 22	21.4	q	14, 17
19		1.31	s		6β, 2'	16.7	q	1, 5, 9, 10, 12, 13
20						163.6	s	
21		6.00	brs		18	98.2	d	22, 23
22		6.29	brs	17	18	122.7	d	17, 21, 23
23						169.8	s	
28		1.35	s			33.6	q	4, 5, 29
29		1.34	s			27.5	q	4, 5, 28
30		1.14	s		11β	16.3	q	7, 8, 9, 14
1'						170.8	s	
2'		2.08	s		19	21.1	q	1'
3-O$\underline{CH_3}$		3.67	s			52.3	q	3

[a] ^1H chemical shift values (δ ppm from $SiMe_4$) followed by multiplicity and then the coupling constants (J/Hz).

[b] Long range ^1H-^{13}C correlation from H to C observed in the HMBC experiment.

Table S4. NMR Spectral Data of **4** in $CDCl_3$.

No.	δ_H[a]	(J in Hz)	^{1}H-^{1}H COSY	NOE	δ_C		HMBC (C)[b]
1	4.03	brd (4.2)	2α, 2β		79.2	d	3
2α	2.66	dd (1.7, 16.8)	1, 2α	19α	35.6	t	3
2β	2.98	dd (4.2, 16.8)	1, 2β				1, 3, 10
3					168.9	s	
4					80.3	s	
5	2.22	m	6α, 6β	9	60.4	d	4, 6, 9, 10, 14, 28
6α	2.47	dd (3.5, 14.6)	5, 6β	28	36.3	t	7, 10
6β	2.84	dd (14.6, 15.8)	5, 6α	19β, 30			5, 7, 10
7					206.1	s	
8					51.1	s	
9	2.50	ddd (3.3, 12.7)		5α	48.0	d	10
10					45.8	s	
11	1.77	m (2H)			18.5	t	
12α	1.40	ddd (7.3, 9.1, 14.4)	11, 12β		28.7	t	11, 13, 14, 18
12β	2.24	m	11, 12α	17			
13					38.6	s	
14					65.7	s	
15	4.12	s		18	53.8	d	14, 16
16					166.0	s	
17	5.43	t (1.5)			75.2	d	13, 14, 18, 20, 21, 22
18	1.18	s		9	20.0	q	12, 13, 14, 17
19α	4.46	d (13.2)		2α, 30	65.1	t	5, 9
19β	4.74	d (13.2)		6β, 29, 30			1, 3, 5, 10
20					133.8	s	
21					168.8	s	
22	7.25	t (1.5)			149.1	d	17, 20, 21, 23
23	5.77	t (1.5)			102.5	d	20, 21, 23-O<u>Me</u>
28	1.29	s		6α	30.2	q	4, 5, 29
29	1.18	s		19β	21.2	q	4, 5, 28
30	1.09	s		6β, 19α, 19β	17.8	q	7, 8, 9, 14
23-O<u>Me</u>	3.60	s			57.8	q	23

[a] ^{1}H chemical shift values (δ ppm from $SiMe_4$) followed by multiplicity and then the coupling constants (J/Hz).

[b] Long range ^{1}H-^{13}C correlation from H to C observed in the HMBC experiment.

Table S5. NMR Spectral Data of **5** in $(CD_3)_2CO$.

No.	δ_H[a]	(J in Hz)	^{1}H-^{1}H COSY	NOE	δ_C		HMBC (C)[b]
1	4.27	dt (1.2, 3.8)	2α, 2β	2α,2β	80.1	d	3, 9, 19
2α	2.87	dd (1.5, 16.7)	1, 2β	1, 19α	36.5	t	1, 3
2β	2.73	dd (4.1, 16.4)	1, 2α	1			1, 3, 10
3					170.0	s	
4					80.7	s	
5	2.60	dd (3.6, 15.9)	6α, 6β	9	59.8	d	4, 6, 7, 9, 10, 19, 28, 29
6α	2.40	dd (3.5, 15.0)	5, 6β	19β, 30	37.1	t	5, 7, 8, 10
6β	3.16	t (5.2)	5, 6α	19β, 30			4, 5, 7, 10
7					208.2	s	
8					51.7	s	
9	2.83	m	11α, 11β	5, 18	48.2	d	8, 10, 11, 12, 19
10					46.7	s	
11α	1.96	m	9, 11β, 12α, 12β		18.8	t	8, 9, 12, 13
11β	2.07	m	9, 11α, 12α, 12β	19α, 30			8, 9, 13
12α	1.44	m	11α, 11β, 12β		29.0	t	11, 13, 14, 18, 23
12b	2.06	m	11α, 11β, 12α	17			12, 17, 18
13					39.9	s	
14					67.6	s	
15	4.19	s		18, 30	55.2	d	14, 16
16					167.3	s	
17	5.35	d (1.2)	22, 23	12β	76.6	d	12, 13, 14, 18, 20, 21, 22
18	1.27	s		9, 22	19.7	s	12, 13, 14, 17
19α	4.65	d (13.5)	19β	2α, 11β, 30	65.7	t	5, 9, 10
19β	4.97	d (13.5)	19α	6β, 29			1, 3, 5, 10
20					129.6	s	
21					173.1	s	
22	7.85	dd (1.7, 2.9)	17, 23	30	154.4	d	17, 20, 21, 23
23	4.99	dd (1.7, 3.5)	17, 22		71.9	t	20, 21, 22
28	1.13	s			21.8	s	4, 5, 28
29	1.24	s		6β, 19β	30.3	s	4, 5, 29
30	1.18	s		11β, 19β	18.2	s	7, 8, 9, 14

[a] ^{1}H chemical shift values (δ ppm from $SiMe_4$) followed by multiplicity and then the coupling constants (J/Hz).

[b] Long range ^{1}H-^{13}C correlation from H to C observed in the HMBC experiment.

Fig. S1. ^{1}H-NMR spectrum of compound **1**.

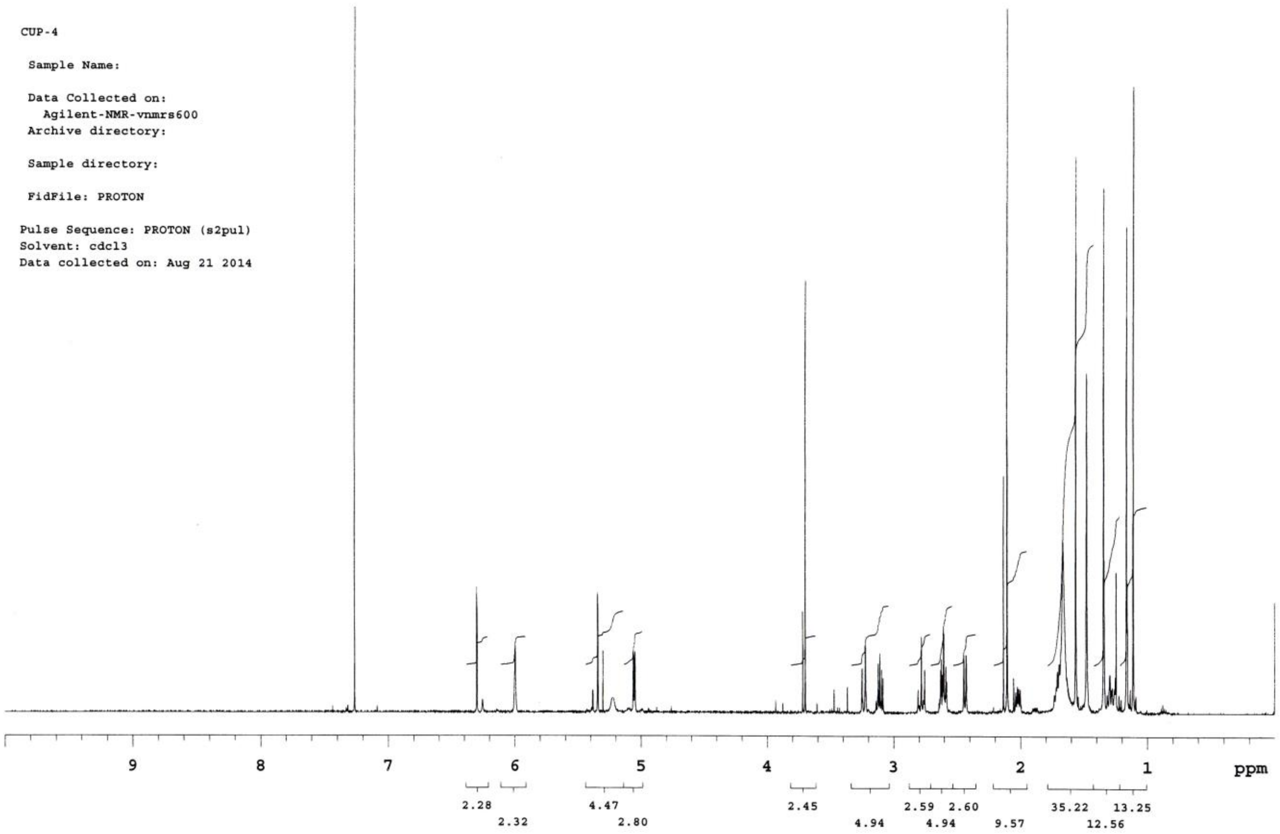

Fig. S2. ^{13}C-NMR spectrum of compound **1**.

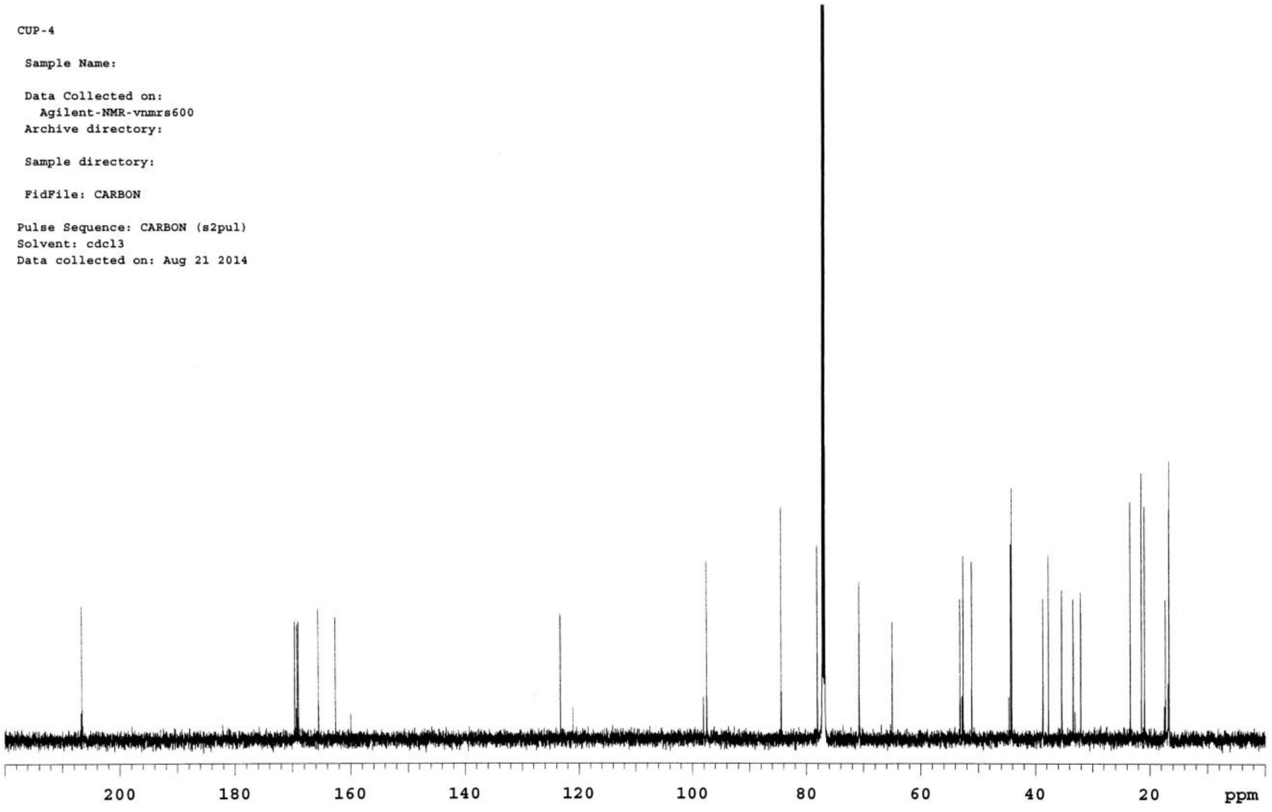

Fig. S3. HSQC spectrum of compound **1**.

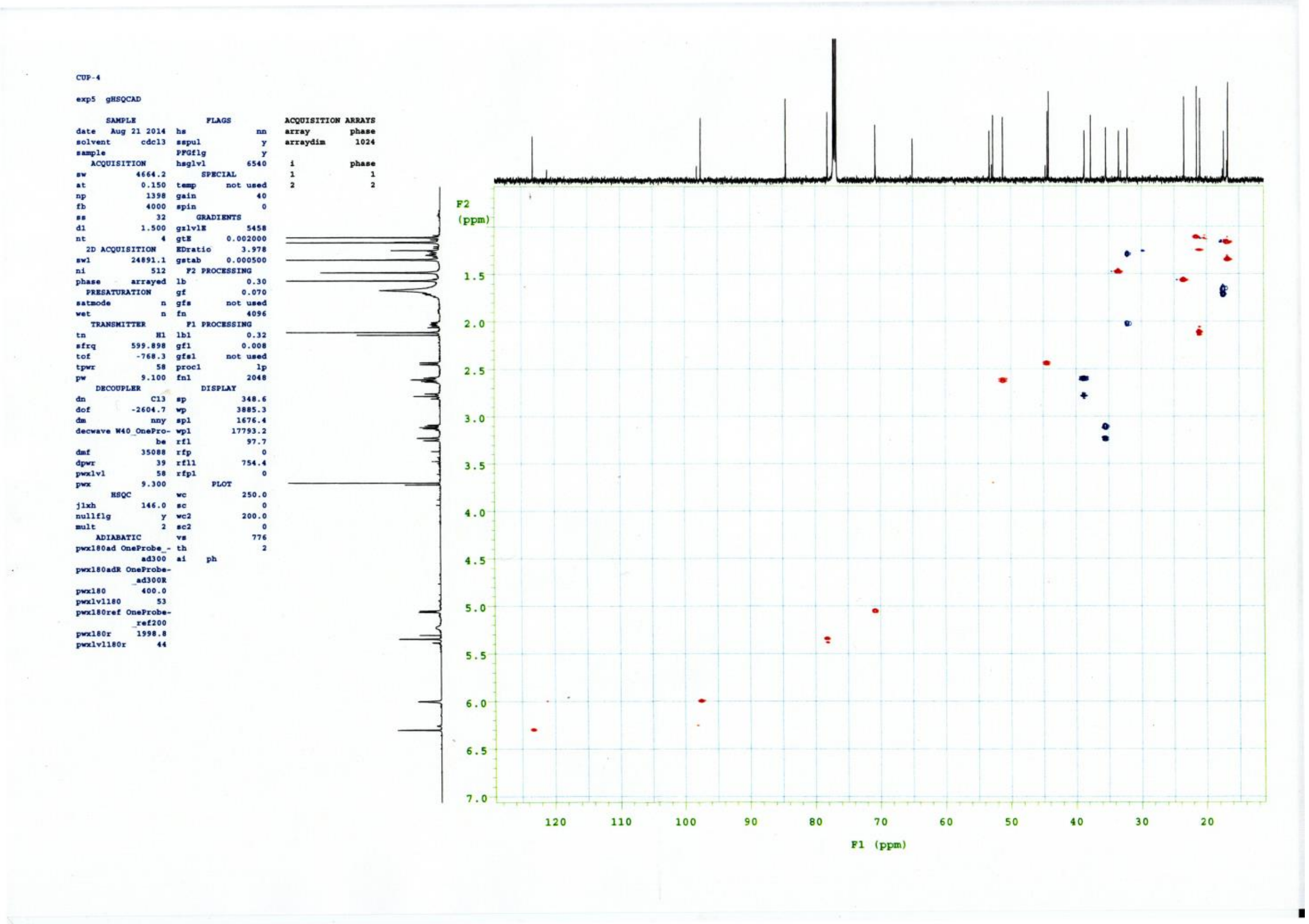

Fig. S4. HMBC spectrum of compound **1**.

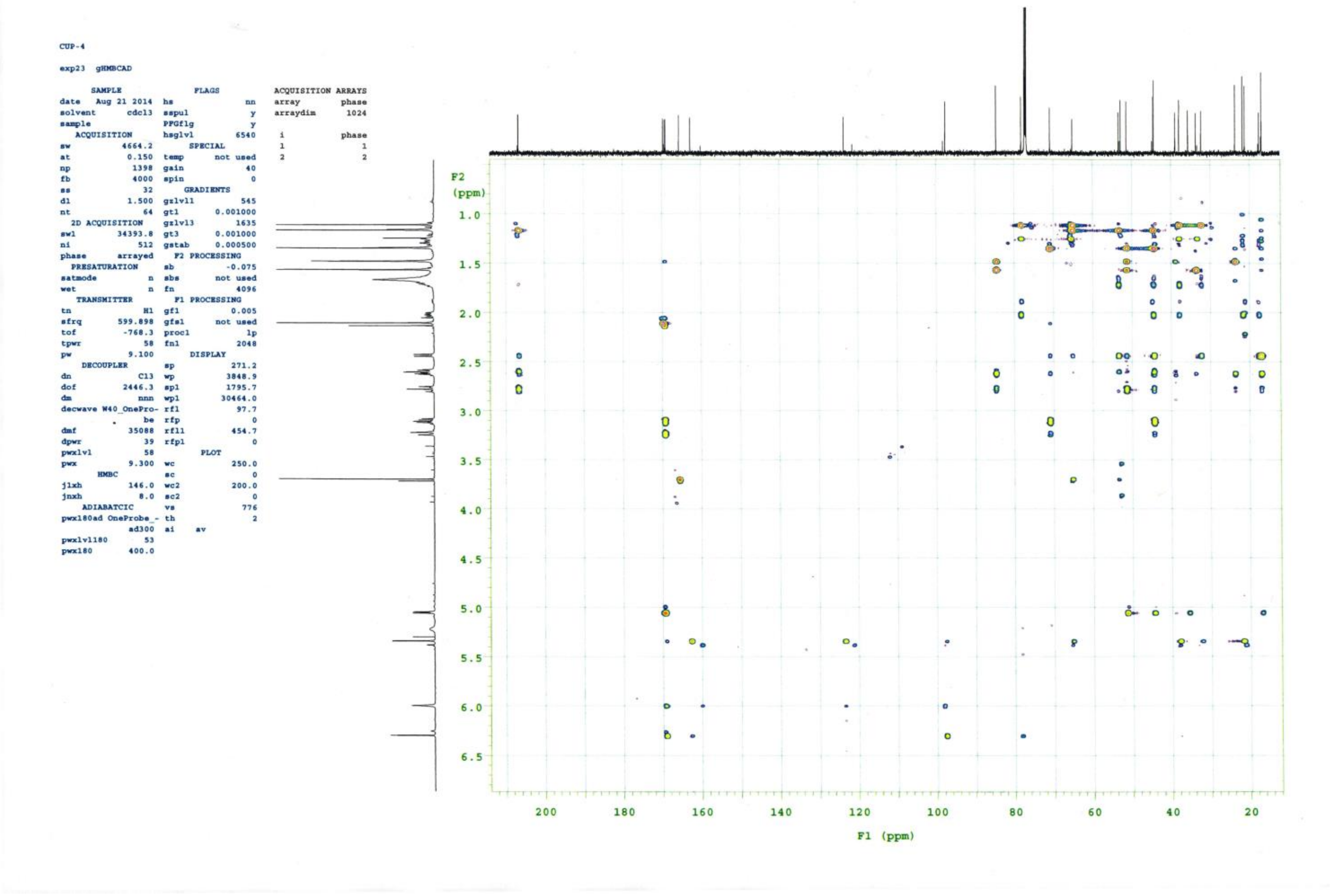

Fig. S5. ^{1}H-^{1}H COSY spectrum of compound **1**.

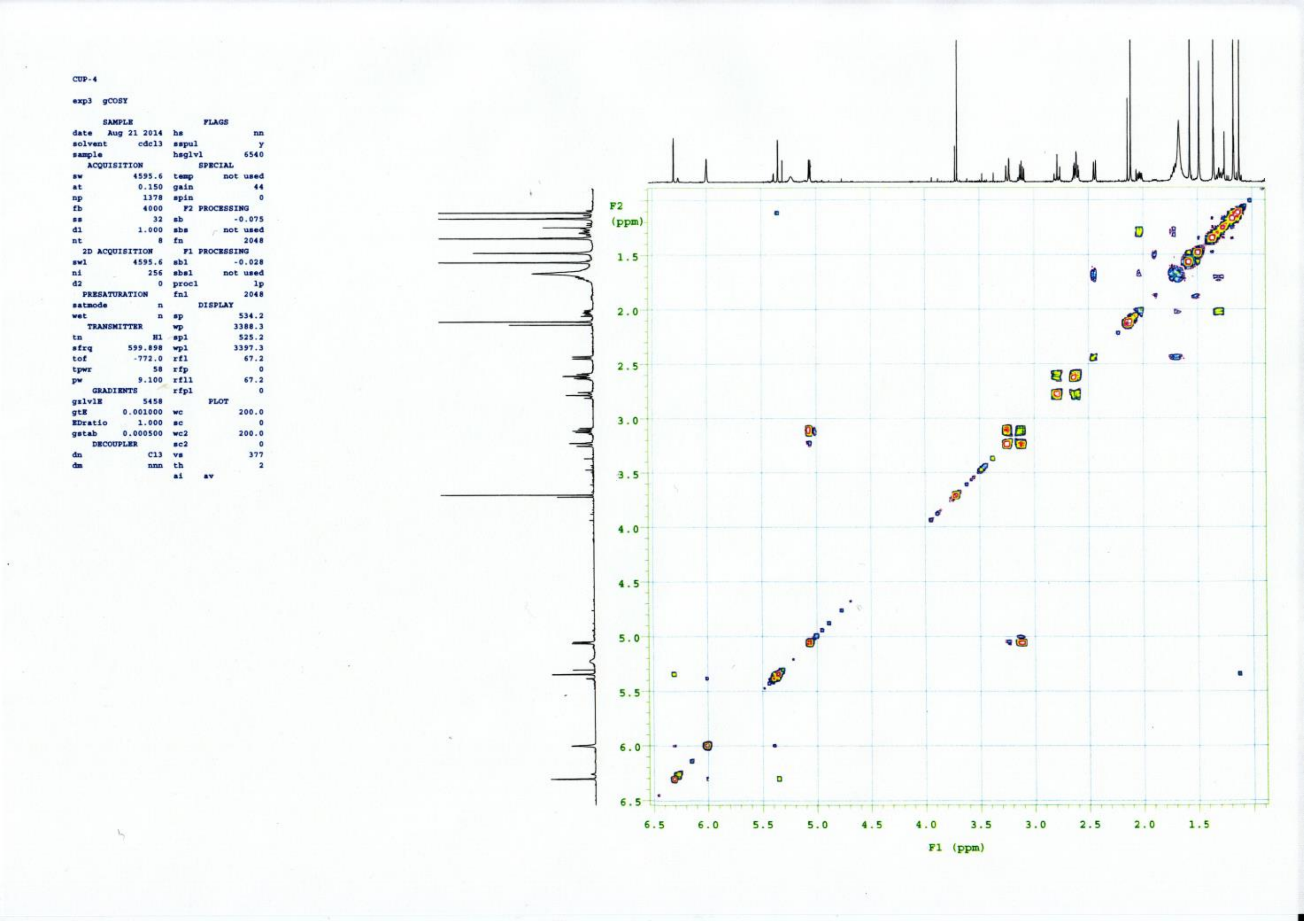

Fig. S6. NOESY spectrum of compound **1**.

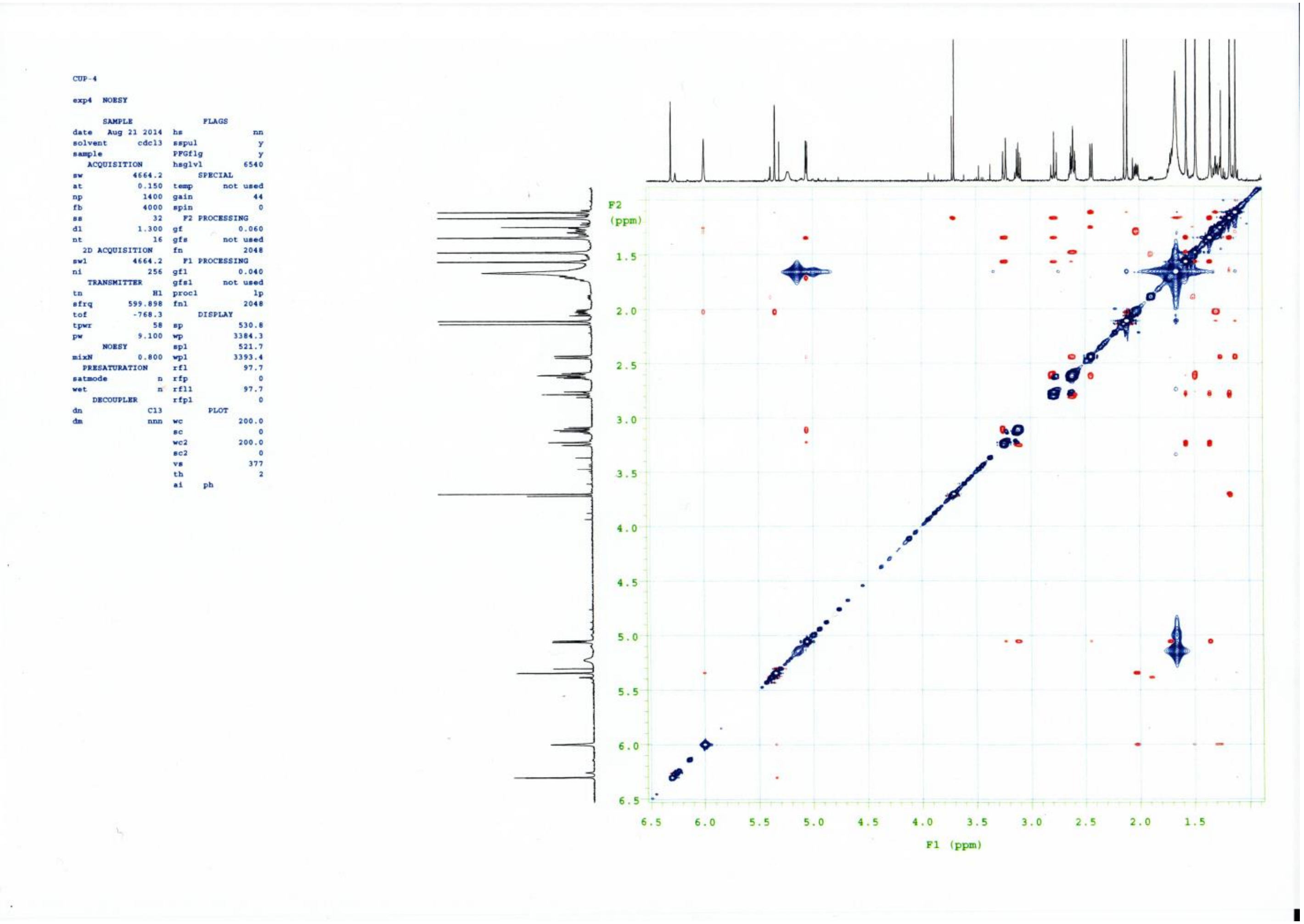

Fig. S7. ^{1}H-NMR spectrum of compound **2**.

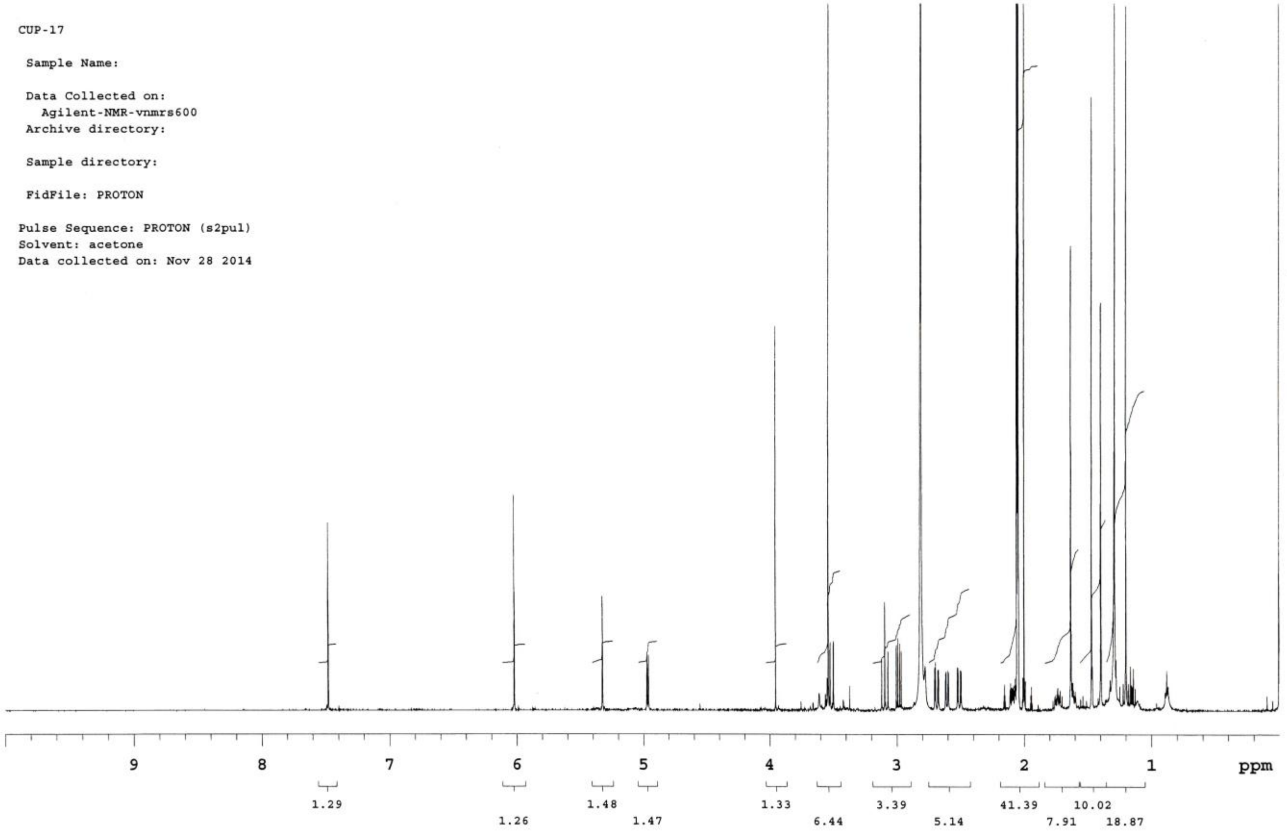

Fig. S8. ^{13}C-NMR spectrum of compound **2**.

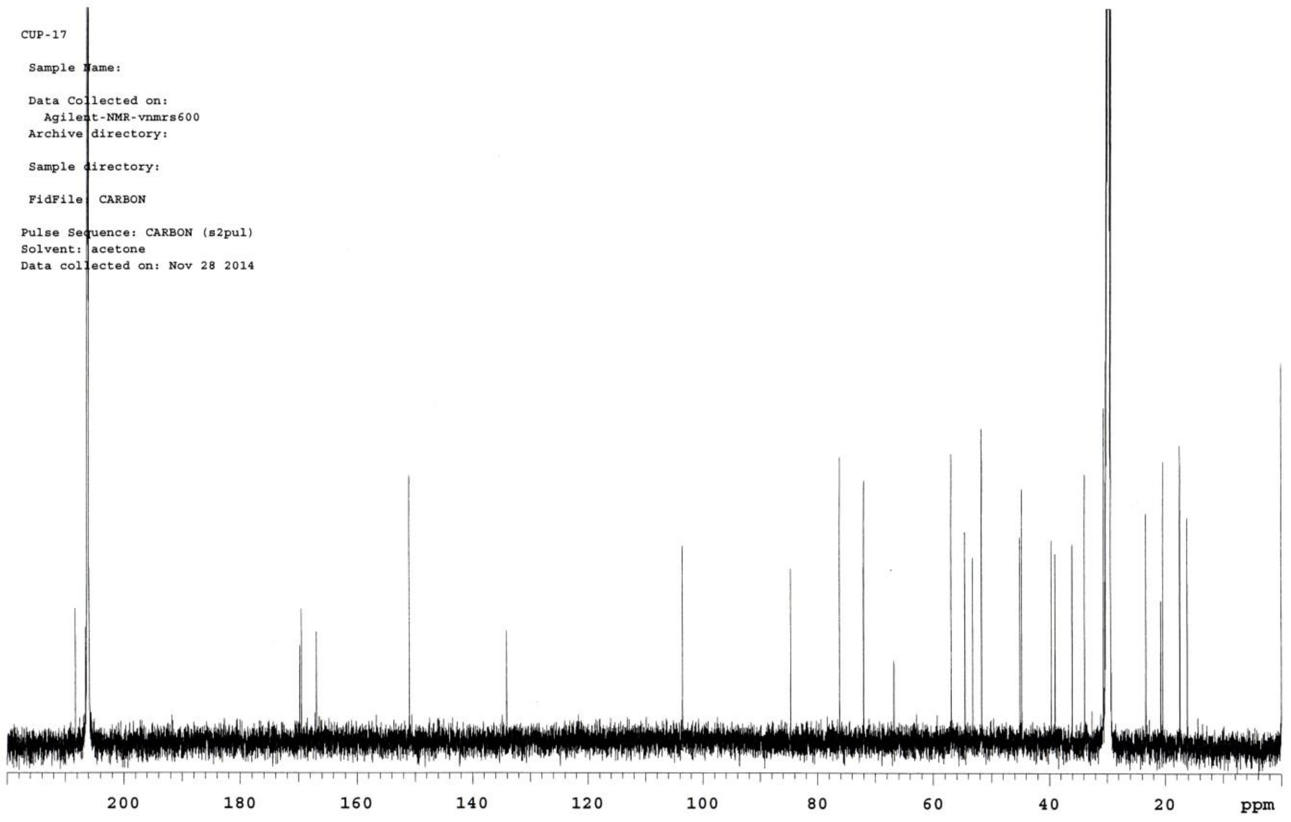

Fig. S9. HSQC spectrum of compound **2**.

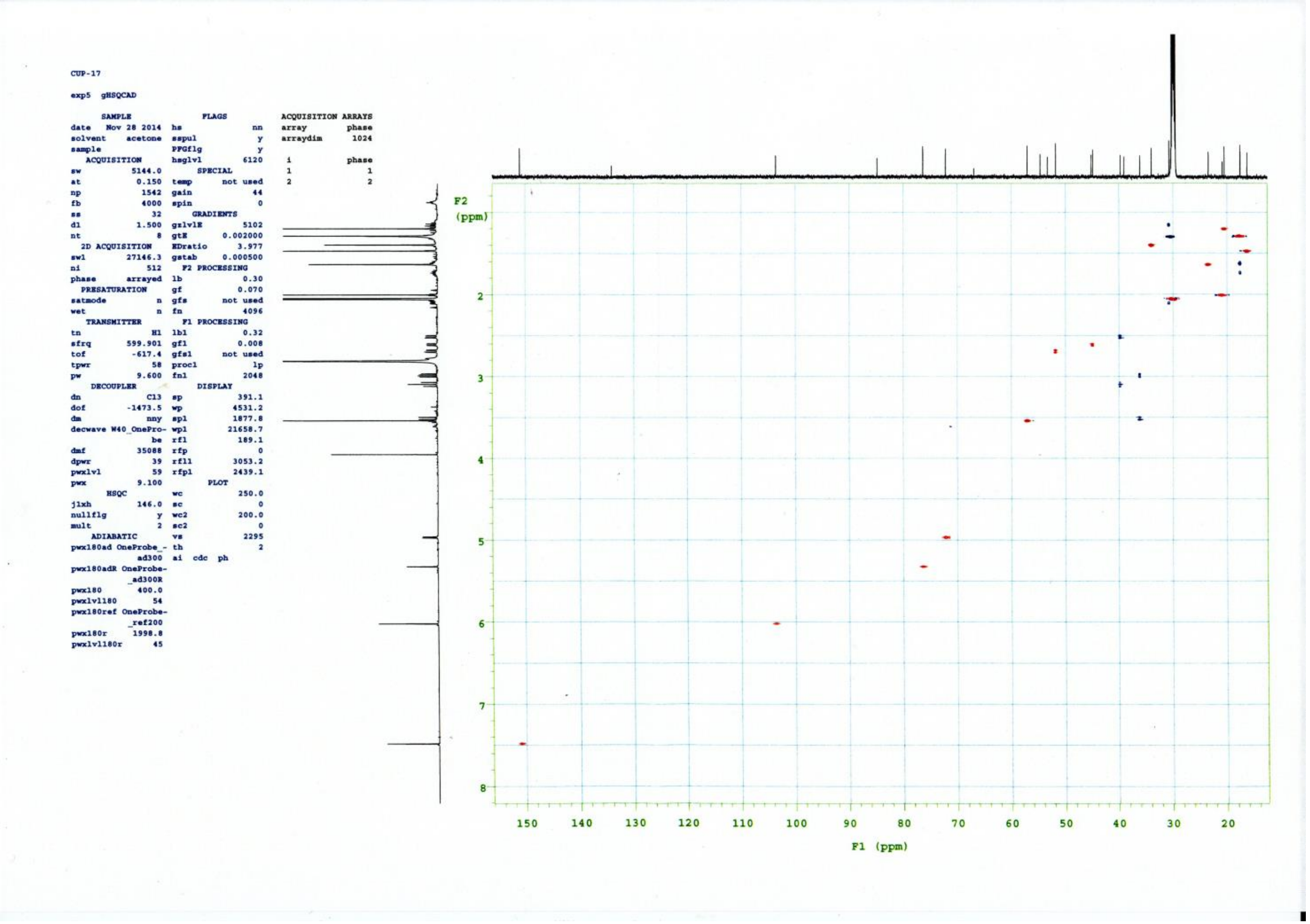

Fig. S10. HMBC spectrum of compound **2**.

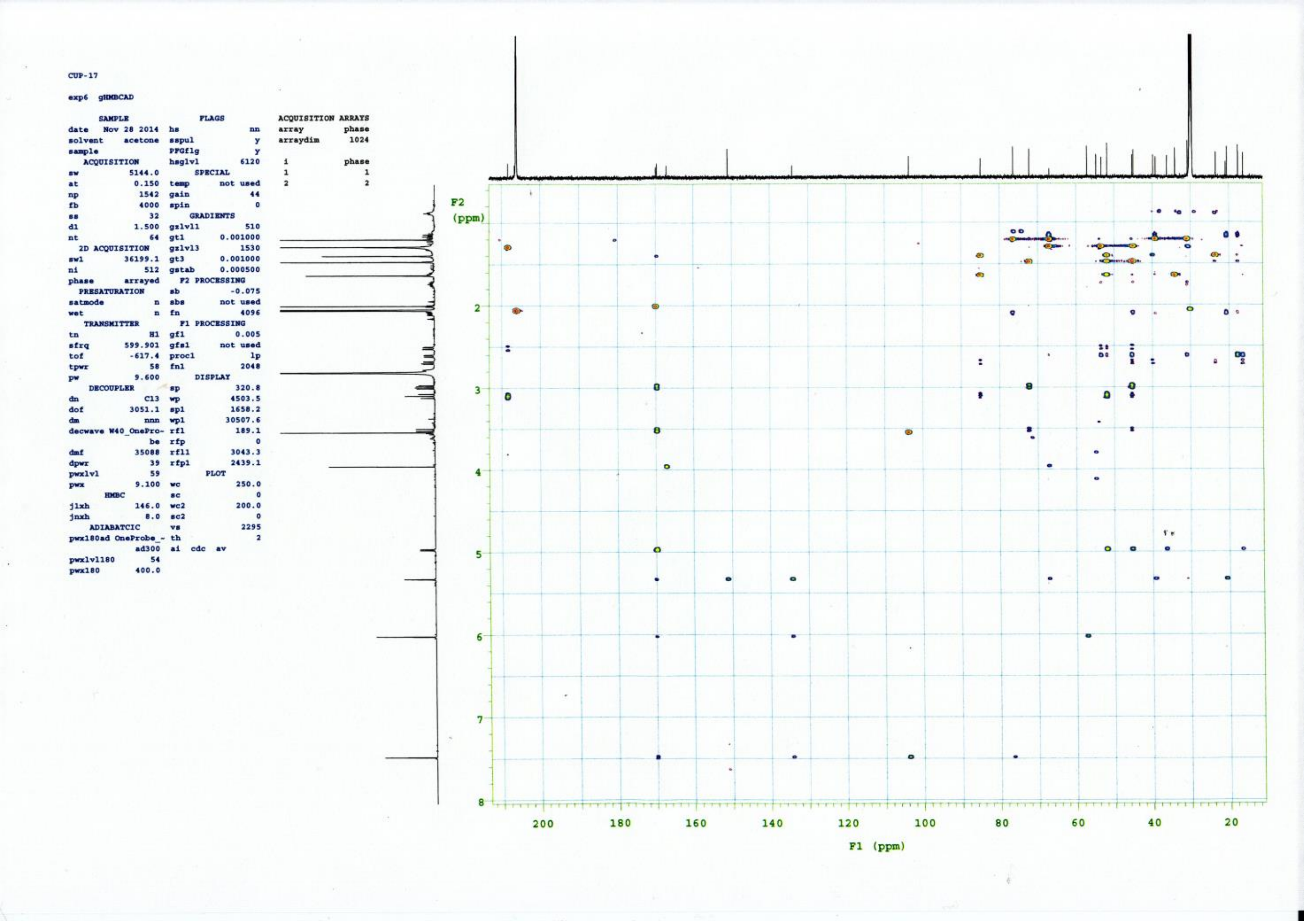

Fig. S11. ^{1}H-^{1}H COSY spectrum of compound **2**.

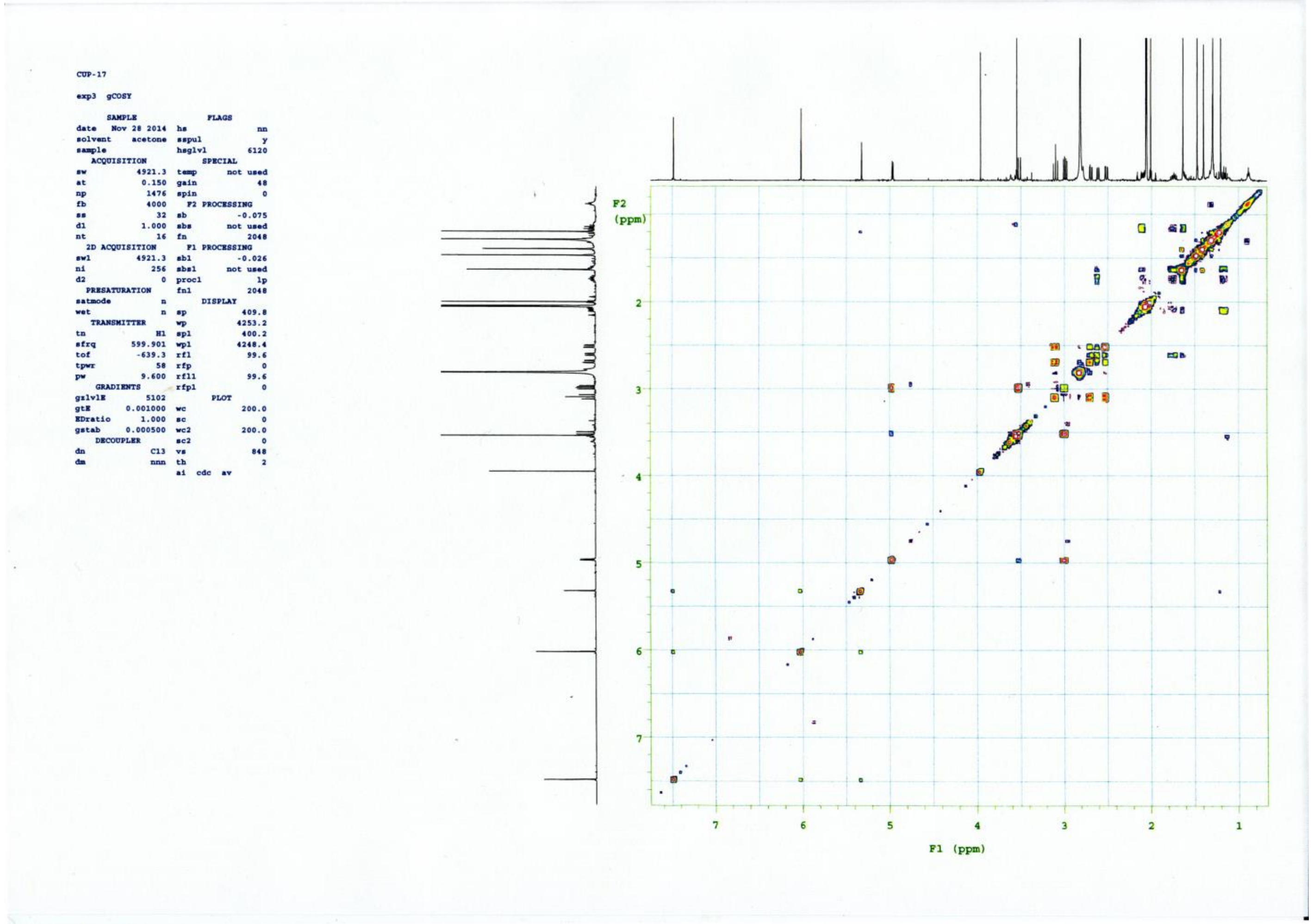

Fig. S12. NOESY spectrum of compound **2**.

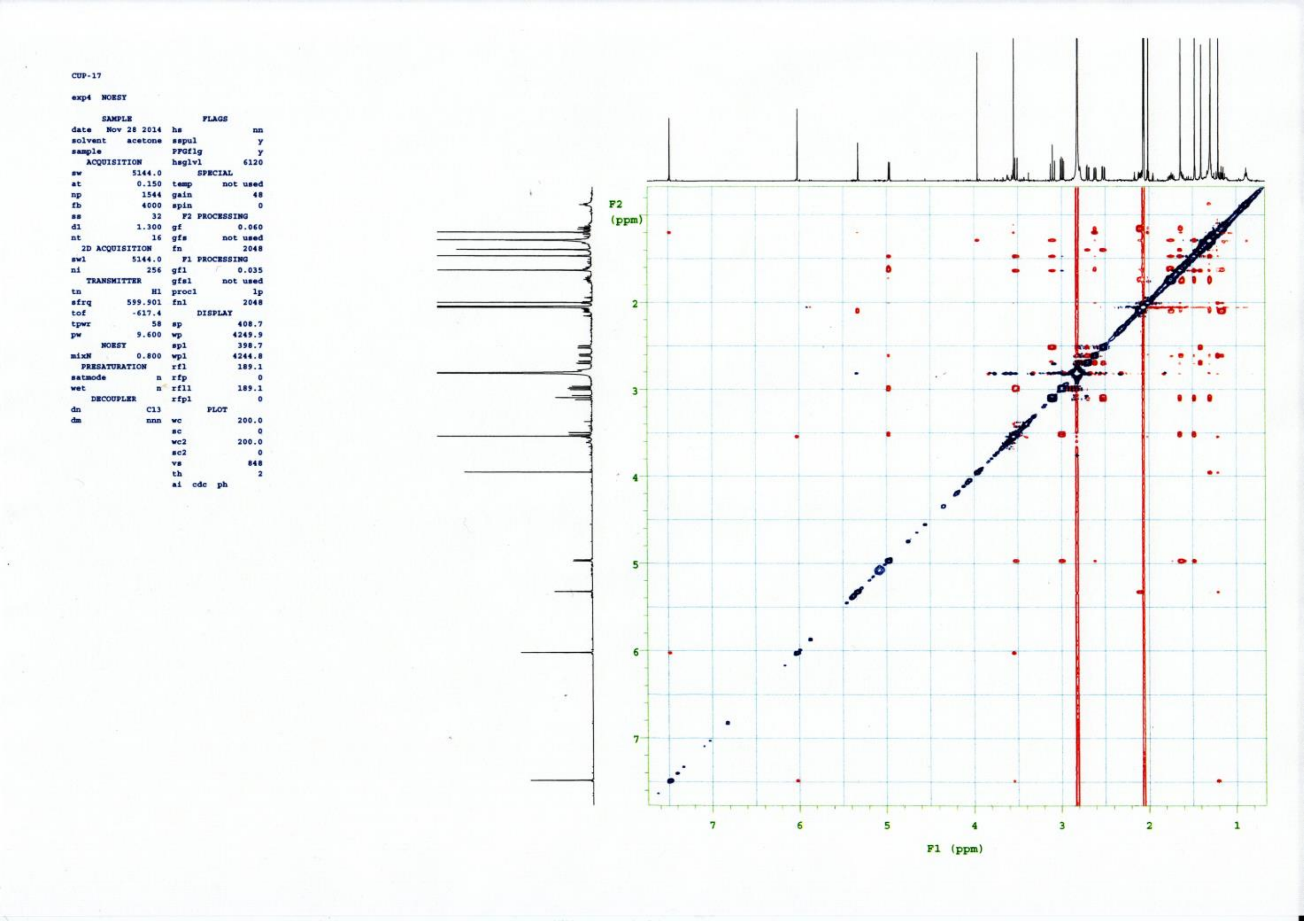

Fig. S13. ^{1}H-NMR spectrum of compound **3**.

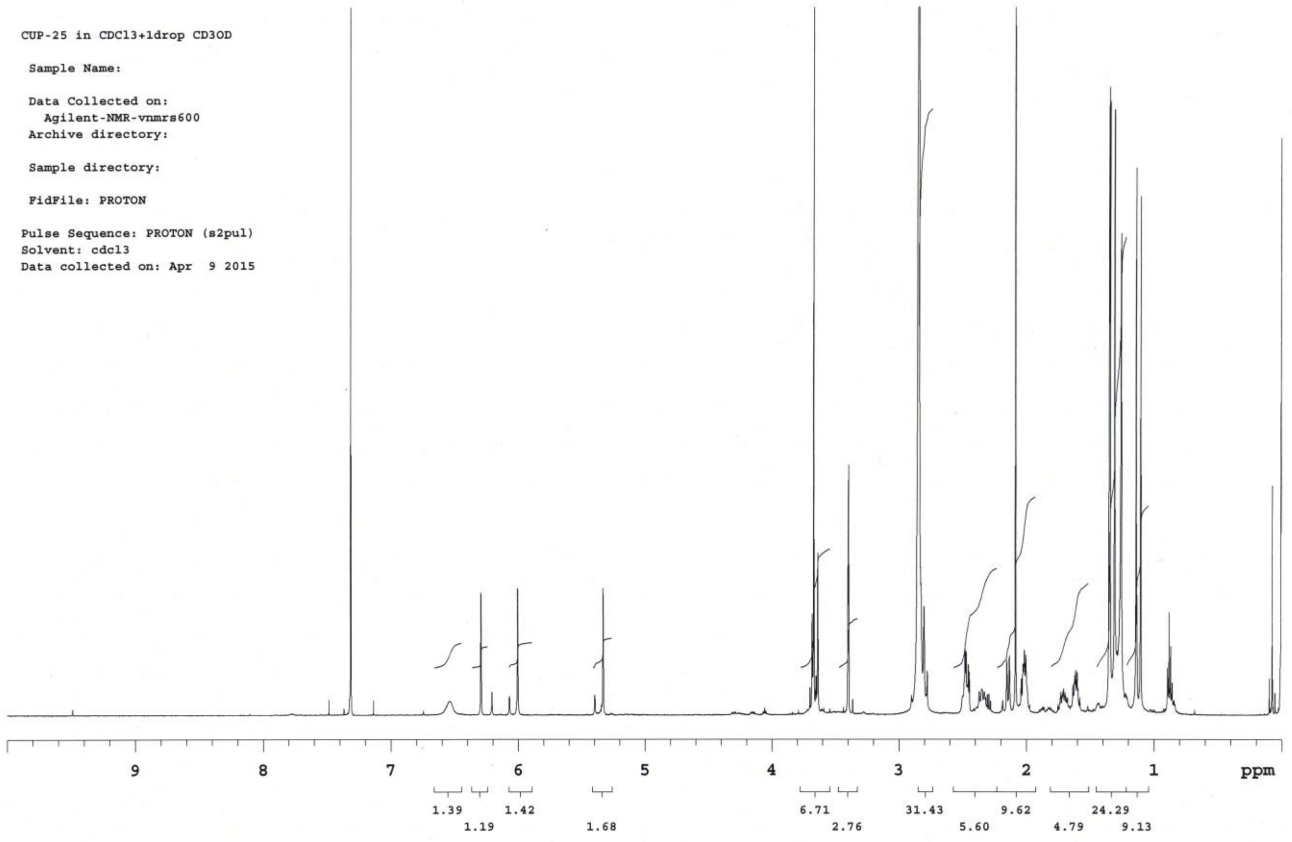

Fig. S14. ^{13}C-NMR spectrum of compound **3**.

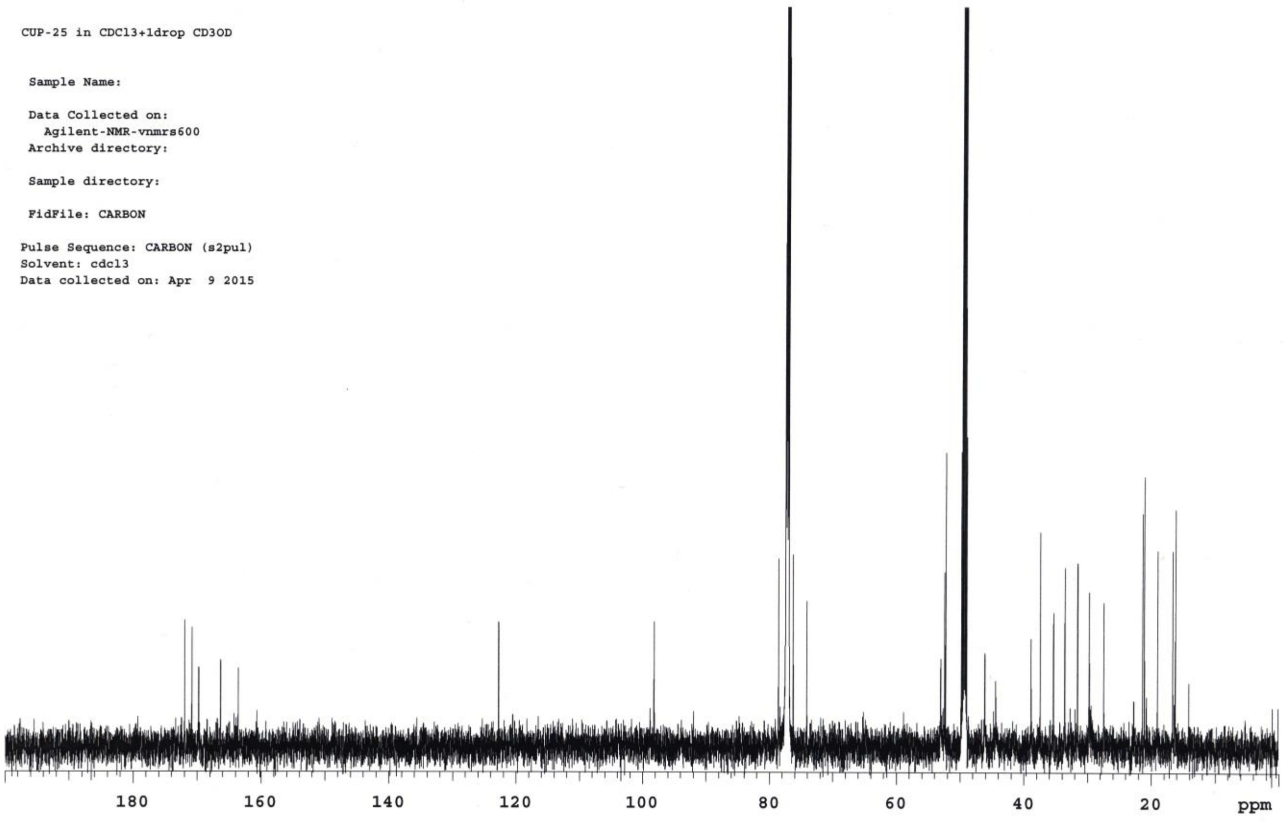

Fig. S15. HSQC spectrum of compound **3**.

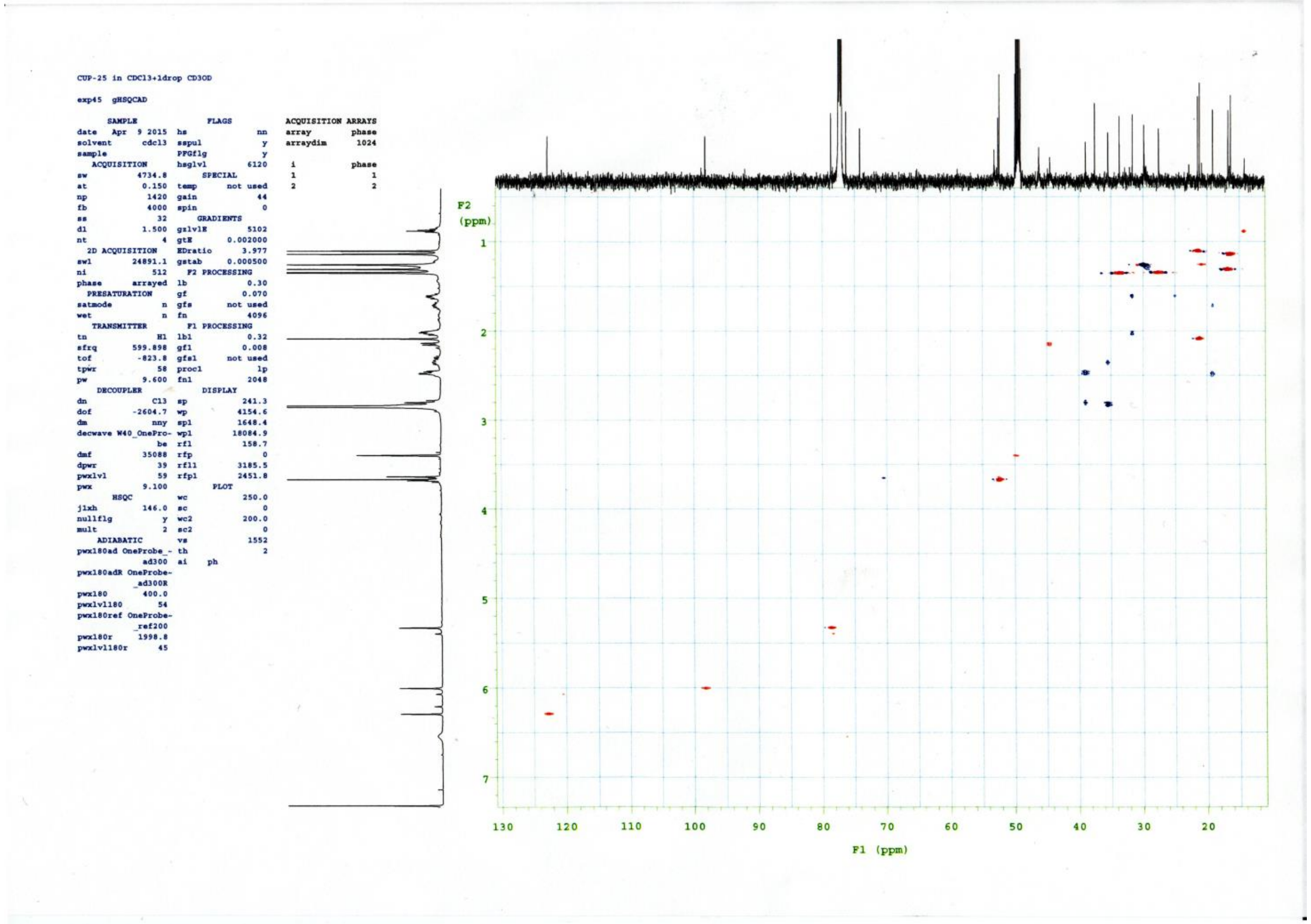

Fig. S16. HMBC spectrum of compound **3**.

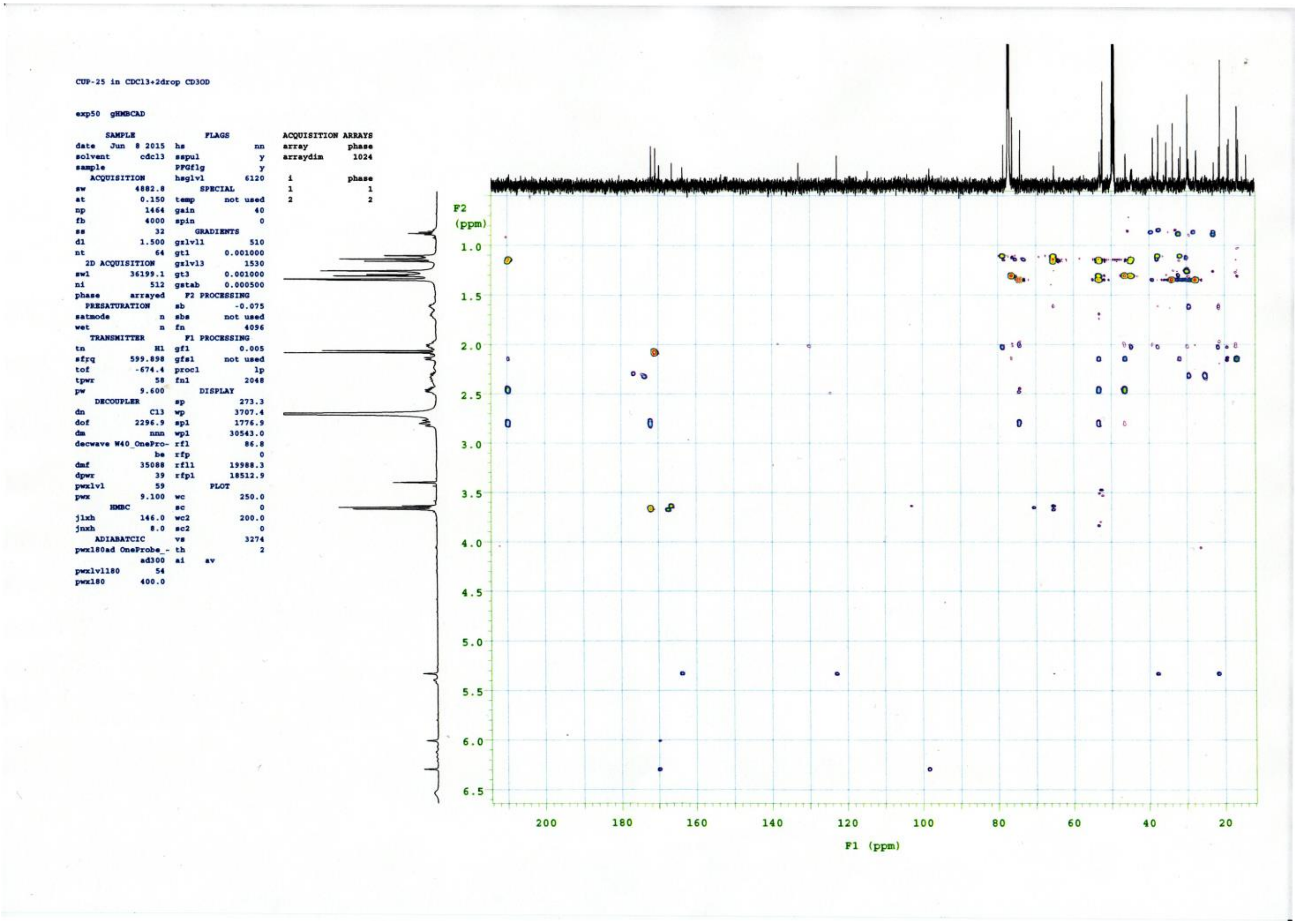

Fig. S17. ^{1}H-^{1}H COSY spectrum of compound **3**.

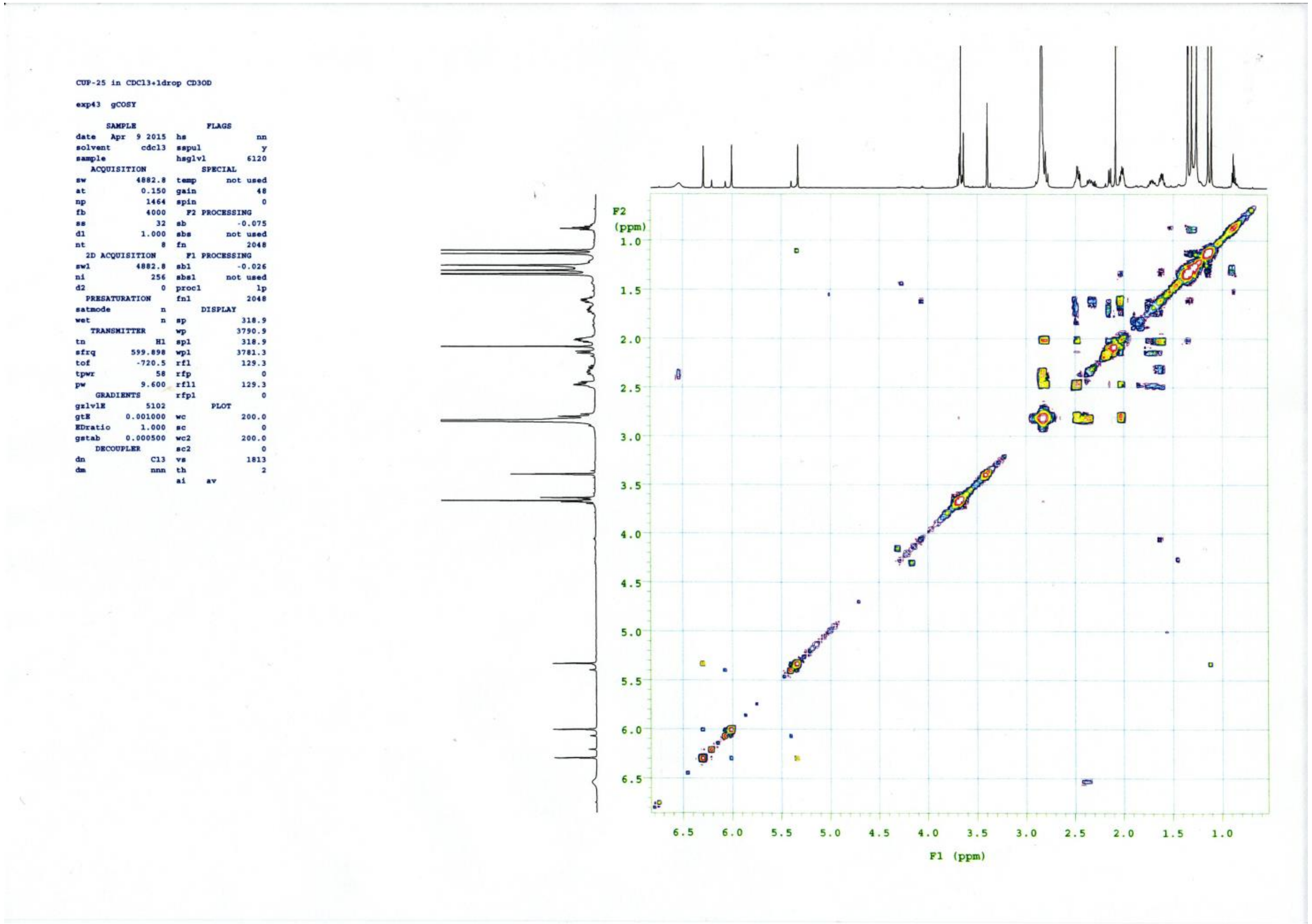

Fig. S18. NOESY spectrum of compound **3**.

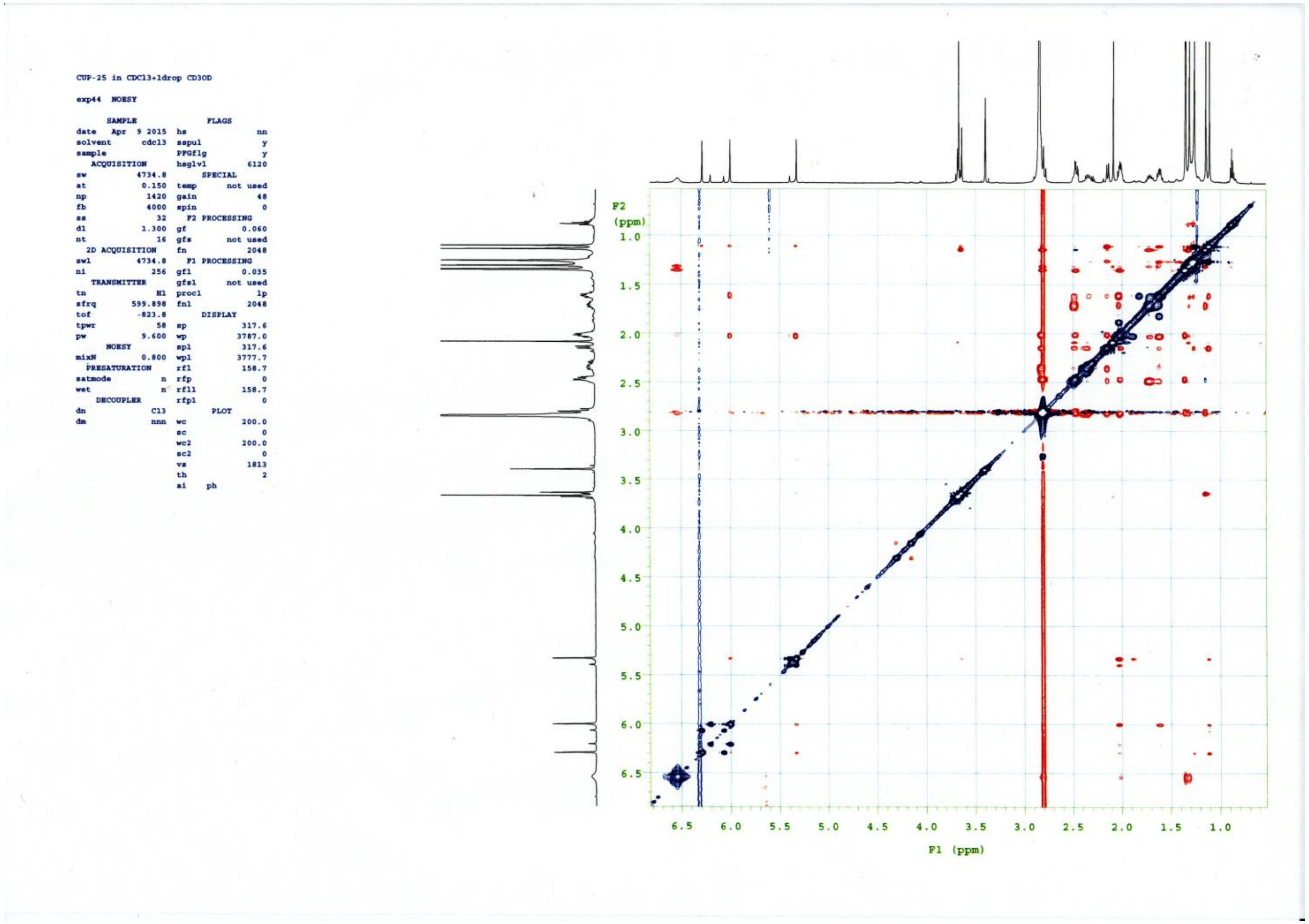

Fig. S19. ^{1}H-NMR spectrum of compound **4**.

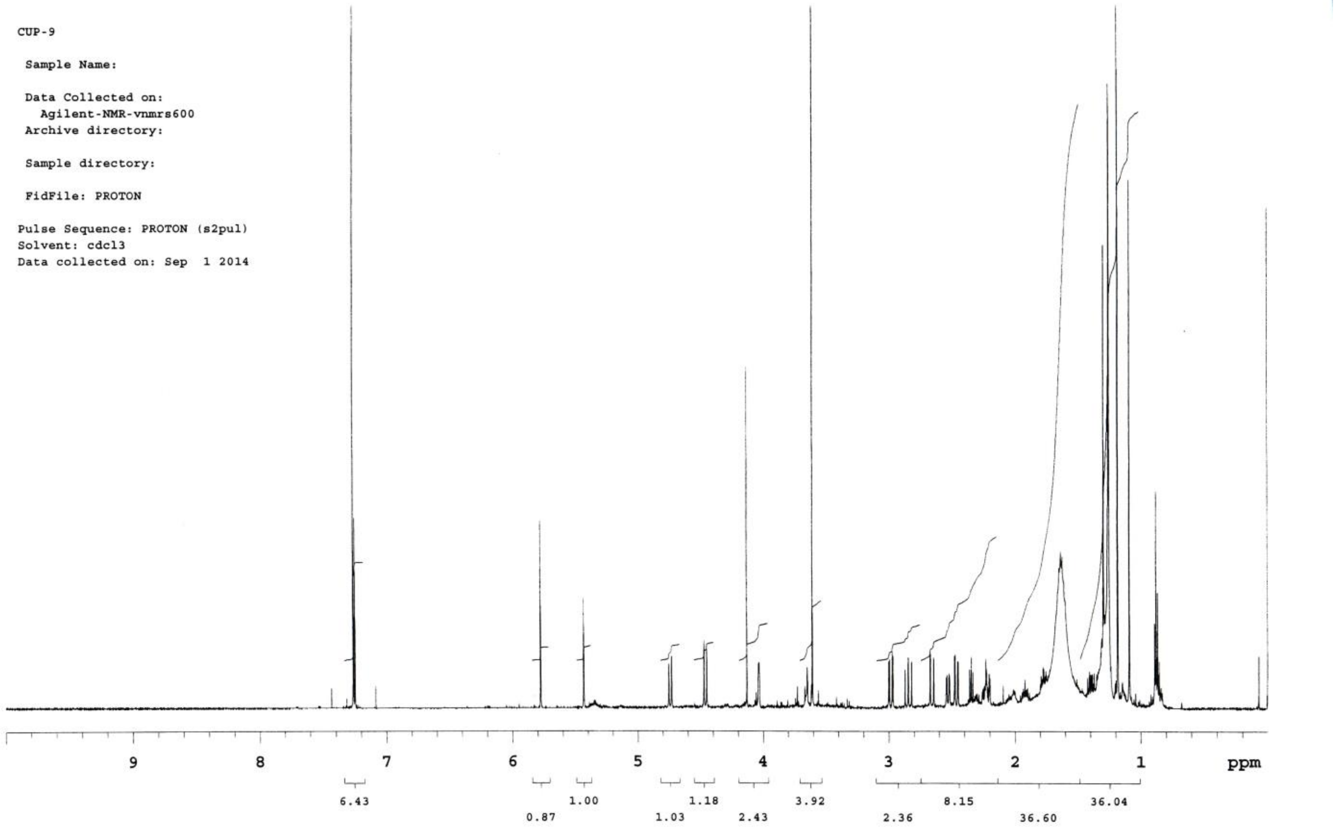

Fig. S20. ^{13}C-NMR spectrum of compound **4**.

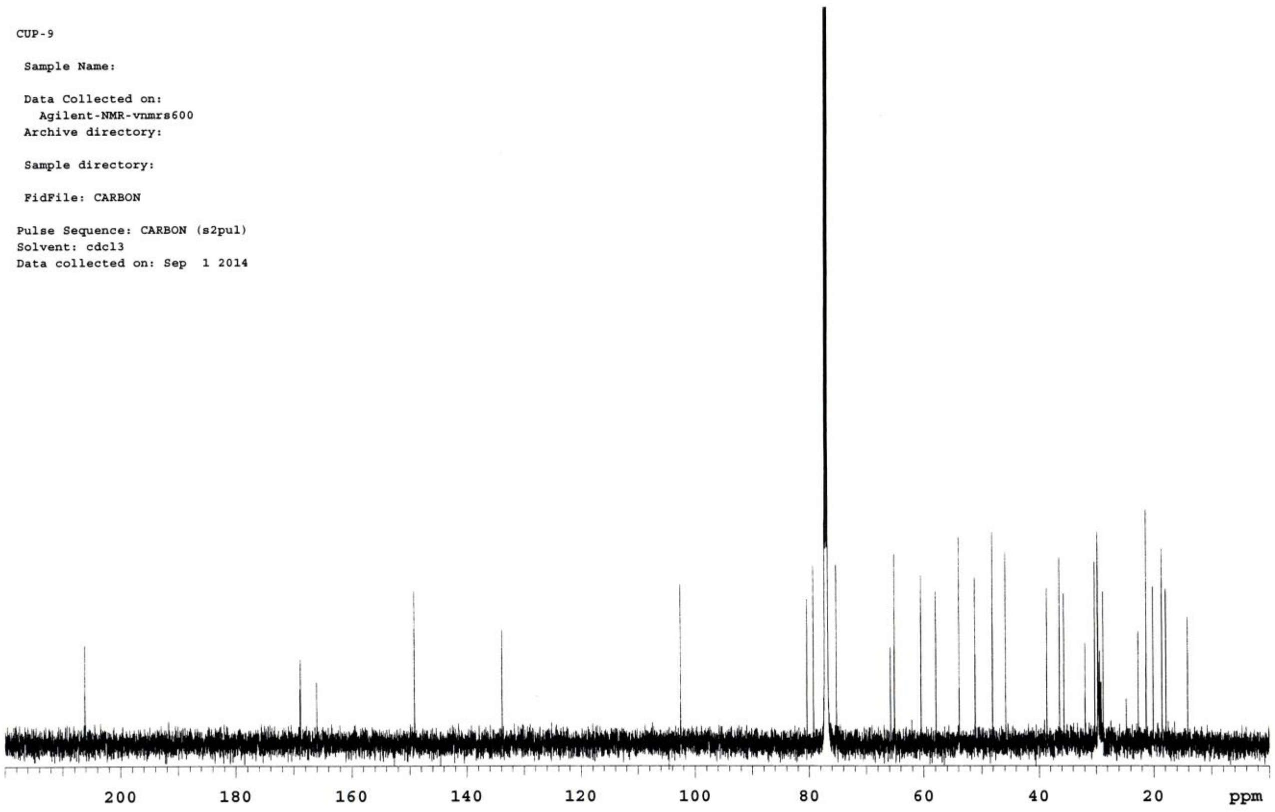

Fig. S21. HSQC spectrum of compound **4**.

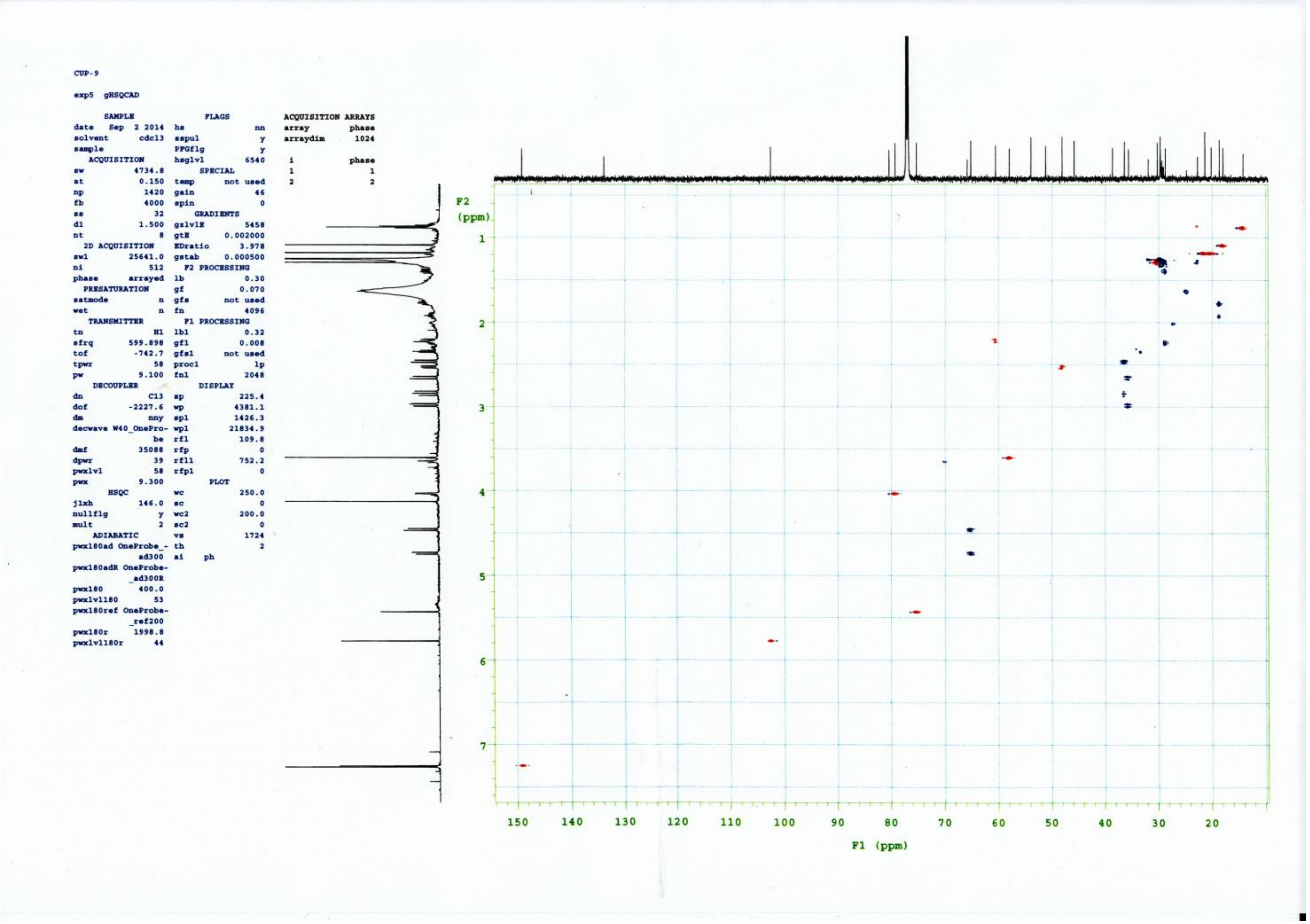

Fig. S22. HMBC spectrum of compound **4**.

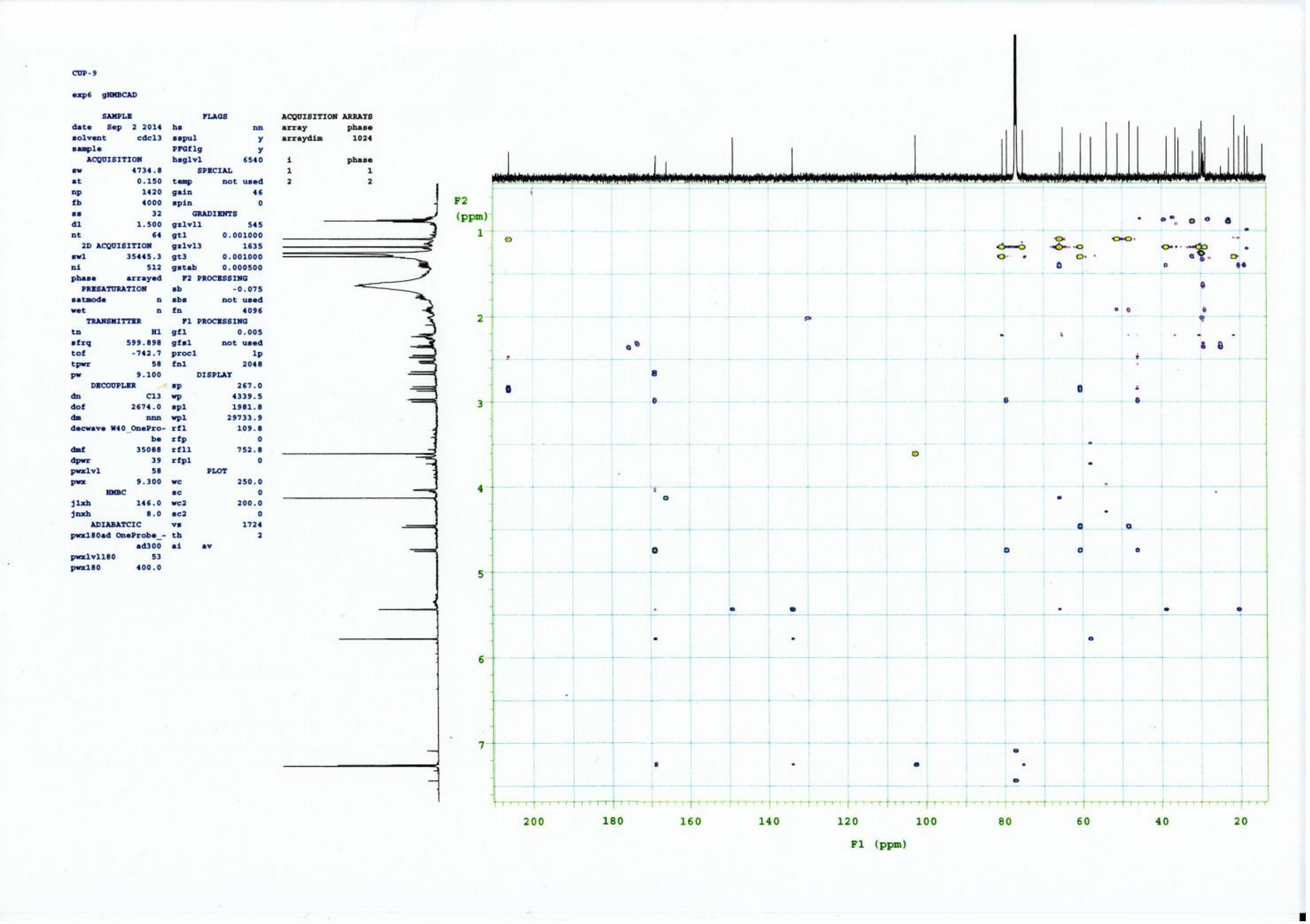

Fig. S23. ^{1}H-^{1}H COSY spectrum of compound **4**.

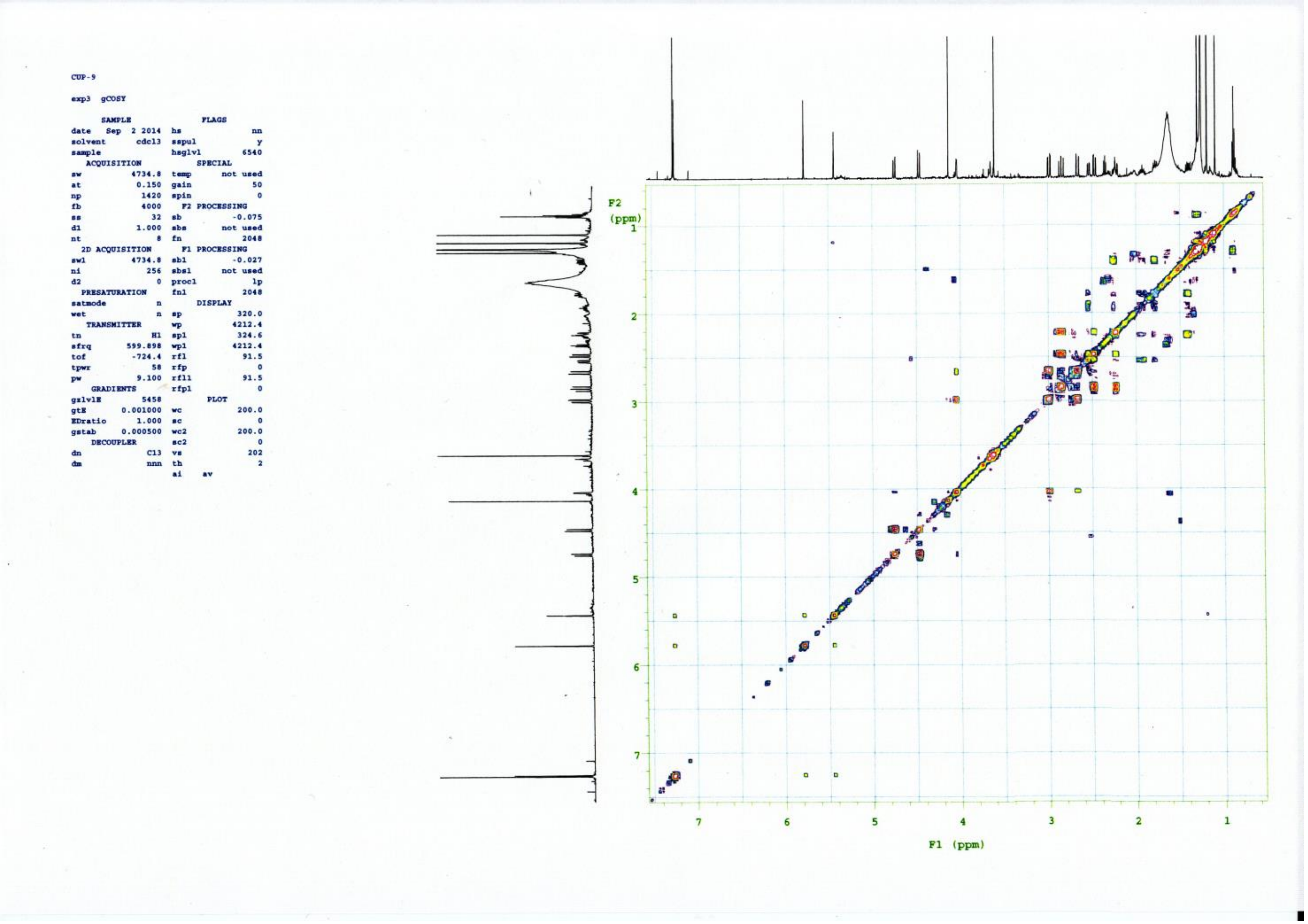

Fig. S24. NOESY spectrum of compound **4**.

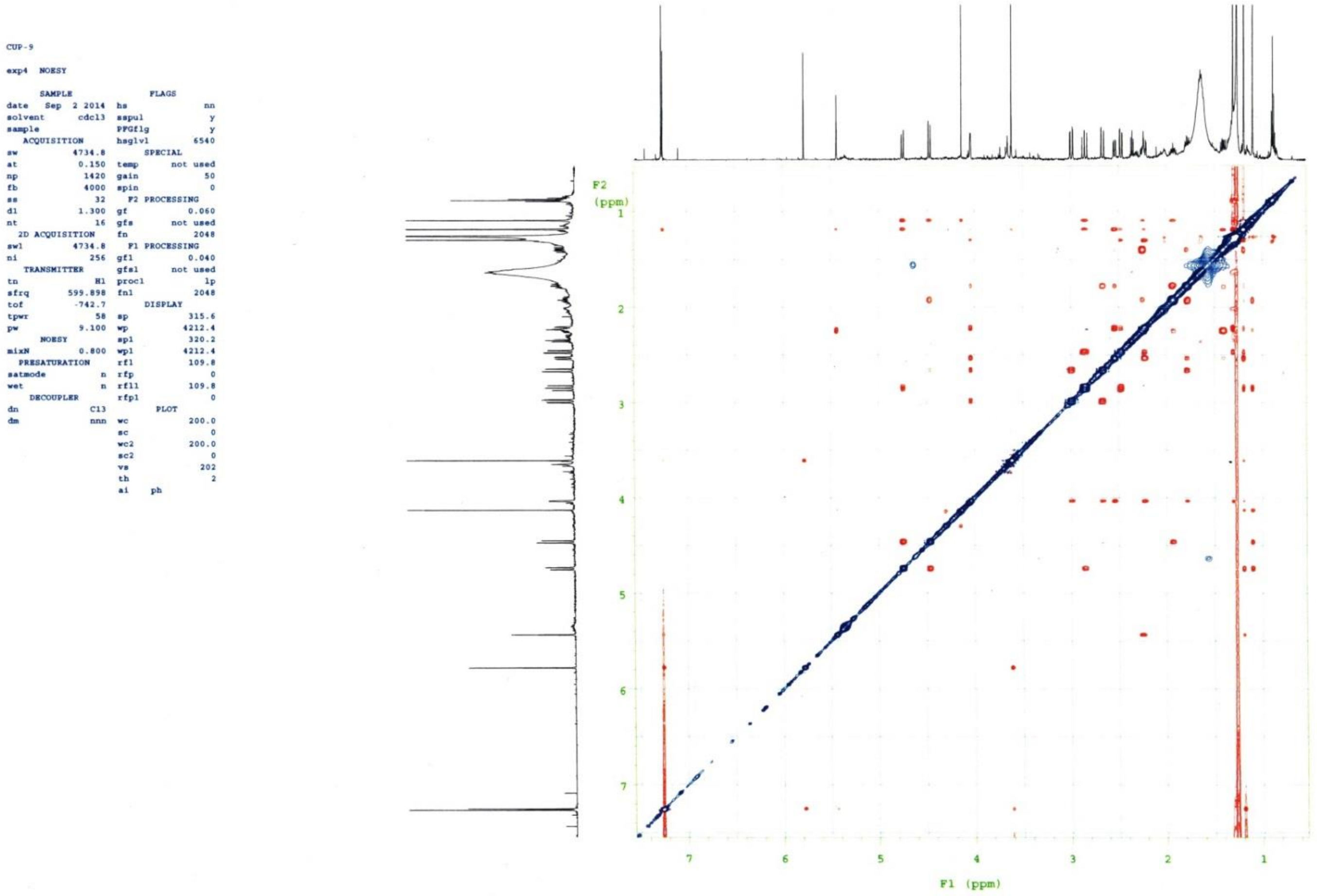

Fig. S25. ^{1}H-NMR spectrum of compound **5**.

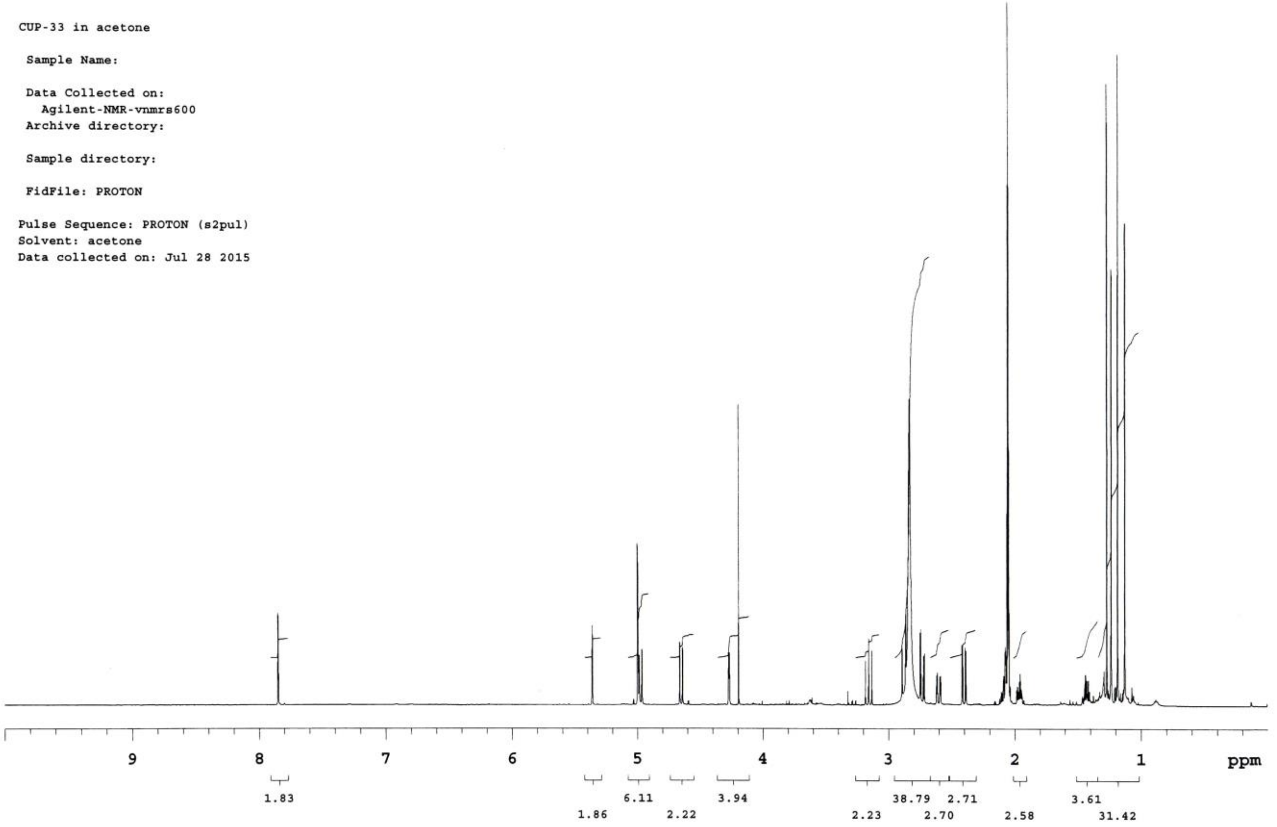

Fig. S26. ^{13}C-NMR spectrum of compound **5**.

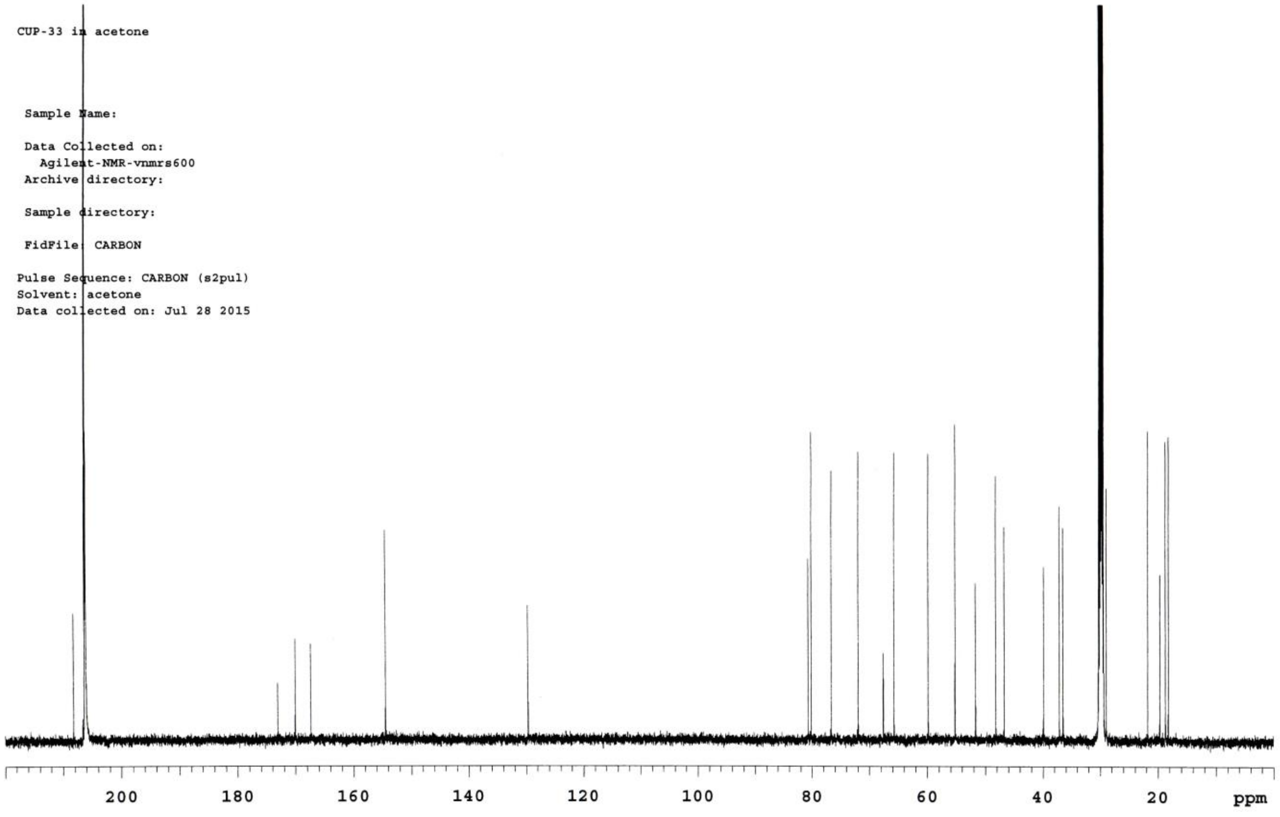

Fig. S27. HSQC spectrum of compound **5**.

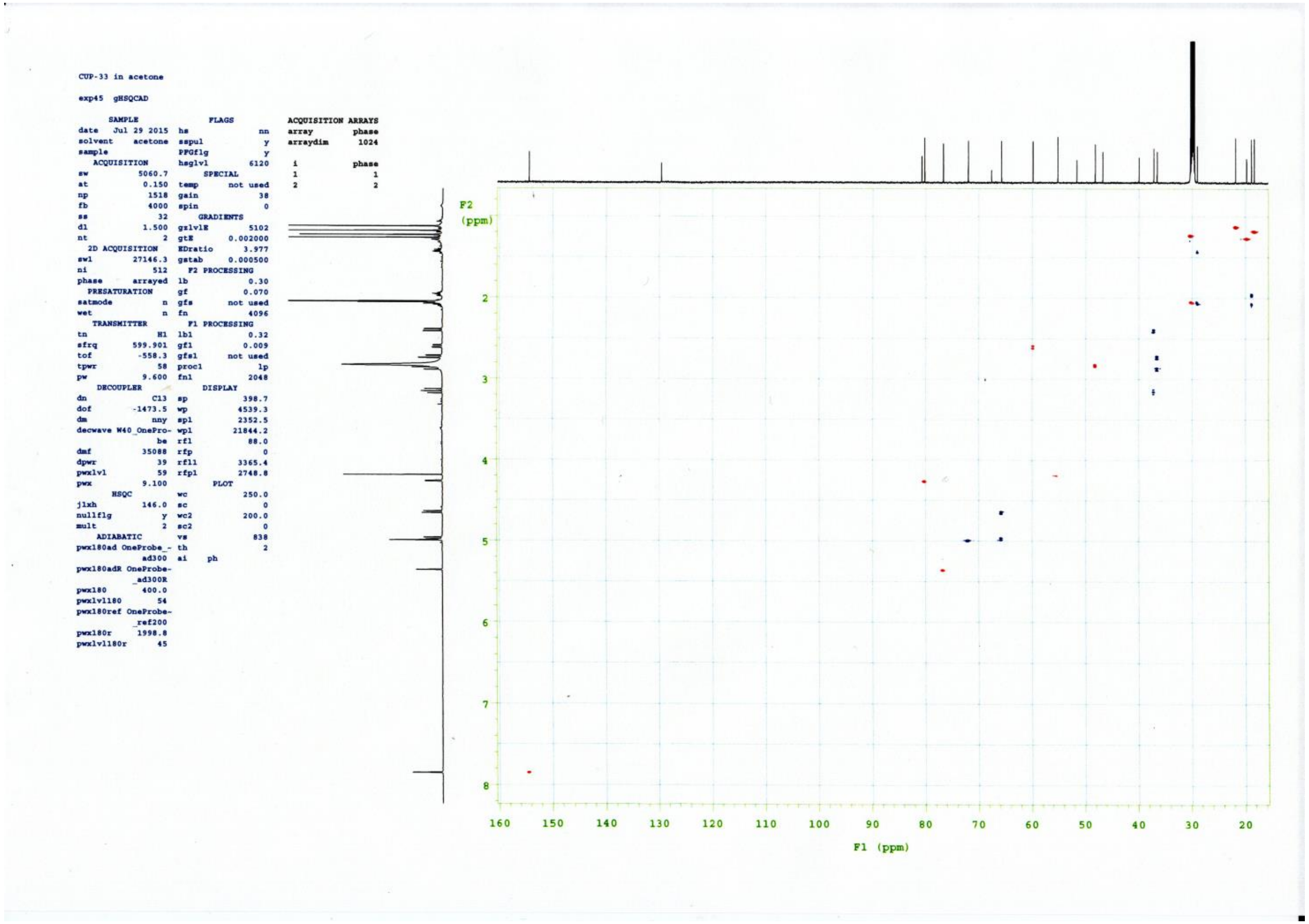

Fig. S28. HMBC spectrum of compound **4**.

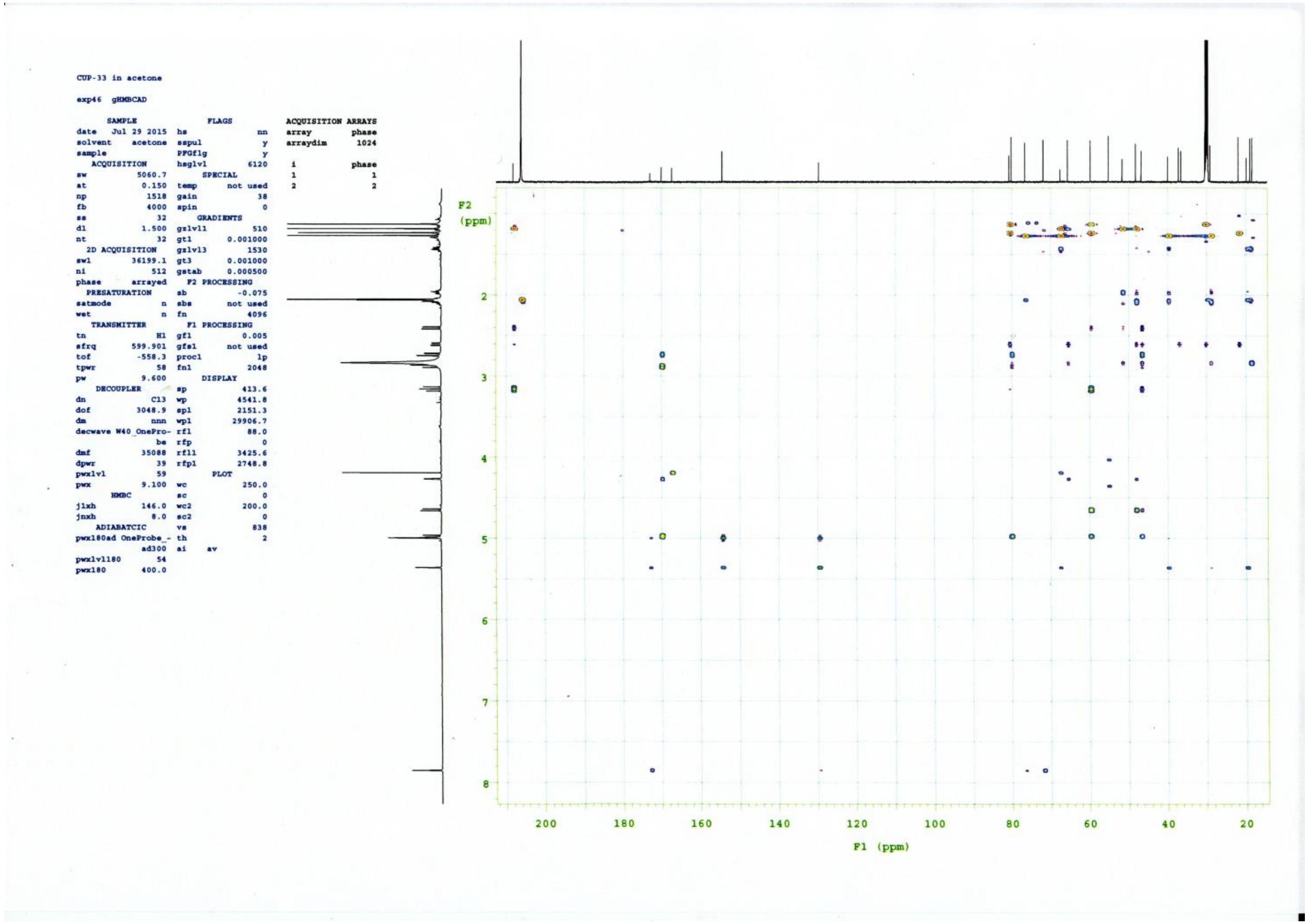

Fig. S29. ^{1}H-^{1}H COSY spectrum of compound **4**.

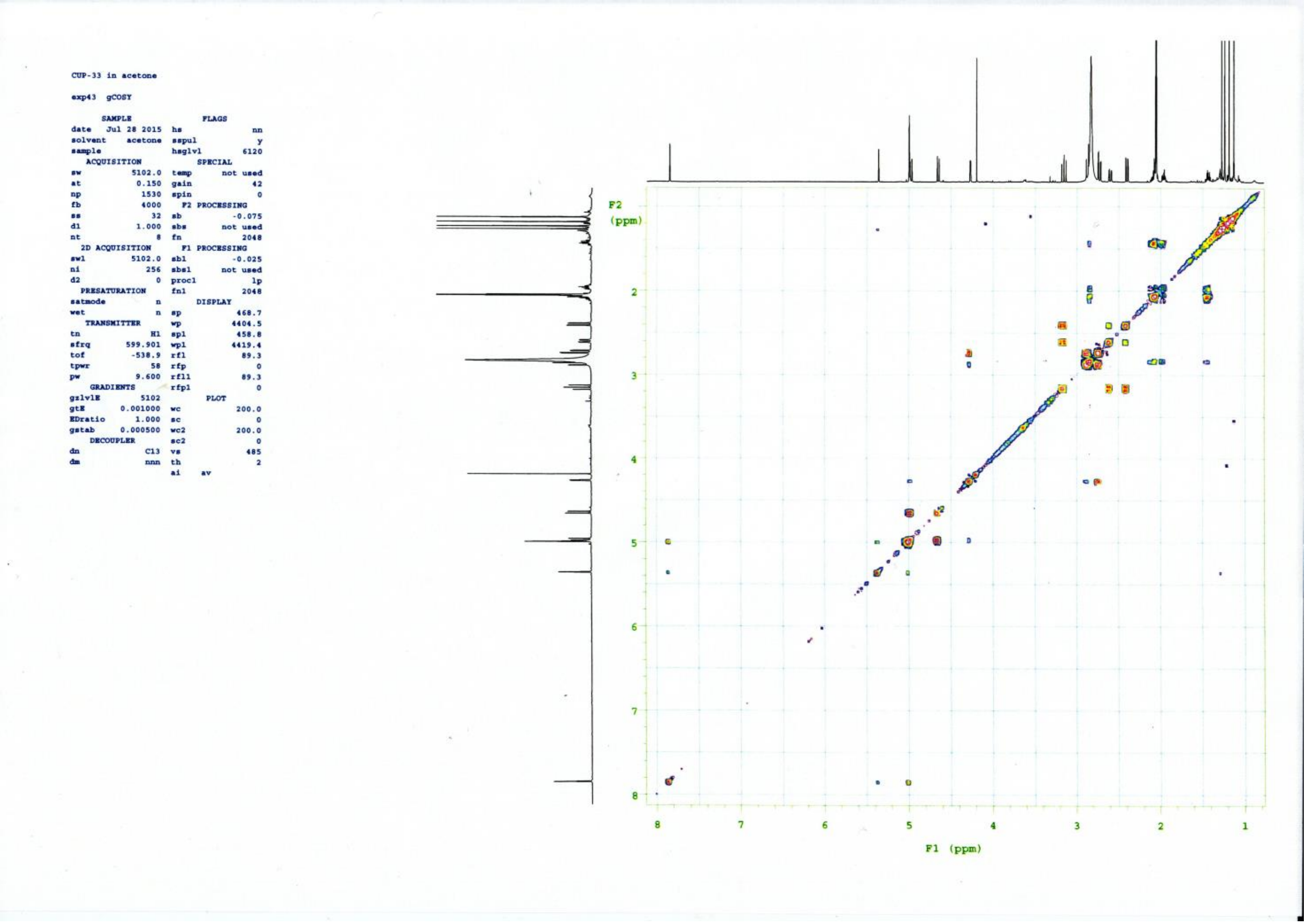

Fig. S30. NOESY spectrum of compound **5**.

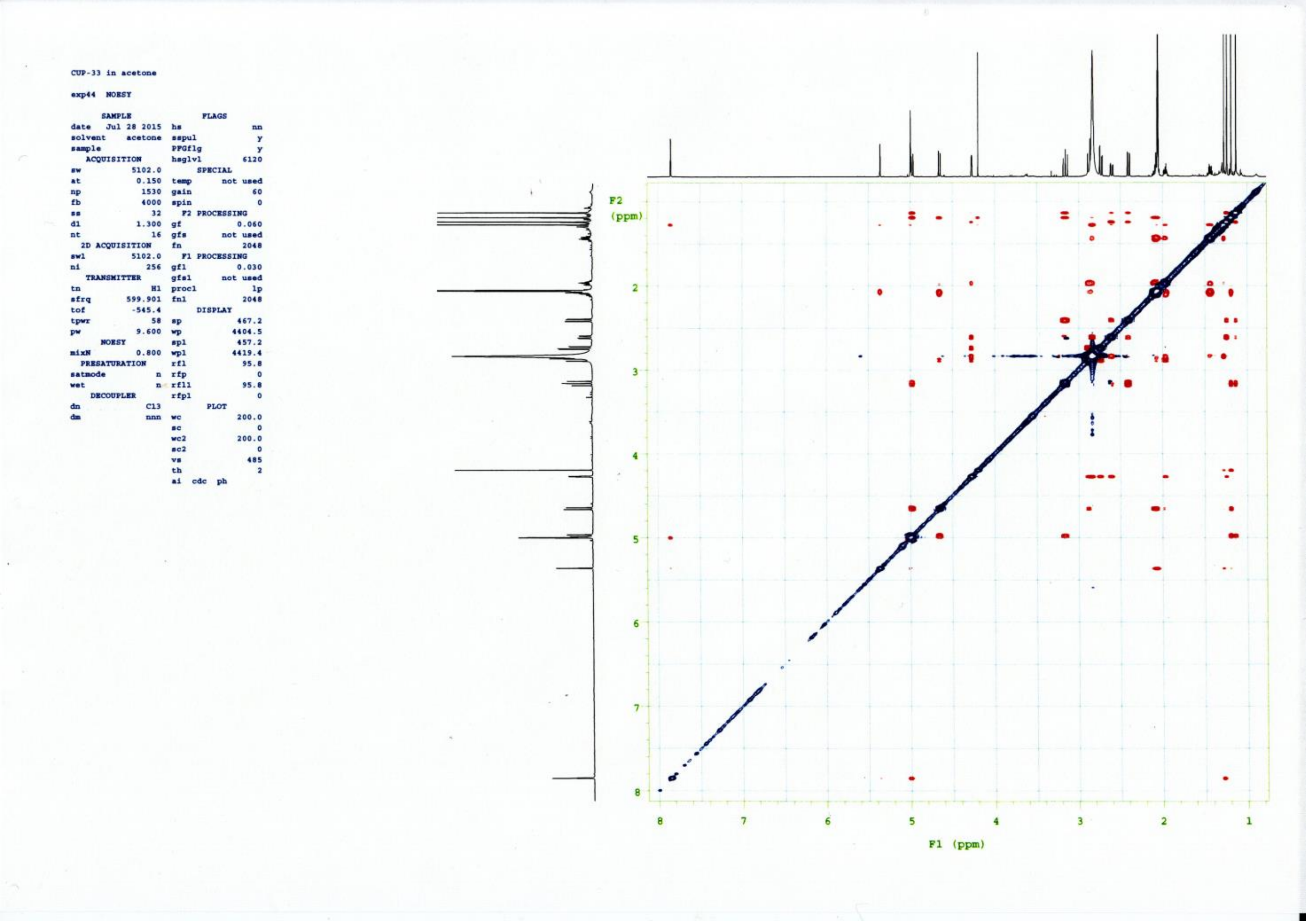

Supplement: Supplementary file 1 [file molecules-22-00907-s001.pdf]
